# Regulation of KDM5C stability and enhancer reprogramming in breast cancer

**DOI:** 10.1038/s41419-022-05296-5

**Published:** 2022-10-03

**Authors:** Qiong Xiao, Chen-Yu Wang, Chuan Gao, Ji-Dong Chen, Jing-Jing Chen, Zhen Wang, Lin-Gao Ju, Shan-Bo Tang, Jie Yao, Feng Li, Lian-Yun Li, Min Wu

**Affiliations:** 1 Frontier Science Center for Immunology and Metabolism, Hubei Key Laboratory of Cell Homeostasis, Hubei Key Laboratory of Developmentally Originated Disease, College of Life Sciences, Renmin Hospital of Wuhan University, Wuhan University, Wuhan, 430072 Hubei China; 2 Department of Biological Repositories, Zhongnan Hospital of Wuhan University, Wuhan, 430071 Hubei China; 3 Department of Medical Genetics, School of Basic Medical Sciences, Wuhan University, 430071 Wuhan, China

**Keywords:** Epigenetics, Breast cancer

## Abstract

Abnormality of enhancer regulation has emerged as one of the critical features for cancer cells. KDM5C is a histone H3K4 demethylase and frequently mutated in several types of cancer. It is critical for H3K4me3 and activity of enhancers, but its regulatory mechanisms remain elusive. Here, we identify TRIM11 as one ubiquitin E3 ligase for KDM5C. TRIM11 interacts with KDM5C, catalyzes K48-linked ubiquitin chain on KDM5C, and promotes KDM5C degradation through proteasome. TRIM11 deficiency in an animal model represses the growth of breast tumor and stabilizes KDM5C. In breast cancer patient tissues, TRIM11 is highly expressed and KDM5C is lower expressed, and their expression is negatively correlated. Mechanistically, TRIM11 regulates the enhancer activity of genes involved in cell migration and immune response by targeting KDM5C. TRIM11 and KDM5C regulate *MCAM* expression and cell migration through targeting H3K4me3 on *MCAM* enhancer. Taken together, our study reveals novel mechanisms for enhancer regulation during breast cancer tumorigenesis and development.

## Introduction

Epigenetic dysregulation is one of the important features for cancer, and critical for chromatin stability, tumorigenesis and metastasis [1–3]. Inhibitors for epigenetic enzymes, such as DNA methyltransferases and histone deacetylases, are developed for clinical therapy [4]. Currently, many inhibitors for histone methyltransferase, demethylases and histone modification readers are under clinical trials or laboratory development [5–7].

With the quick development of Next Generation Sequencing (NGS), epigenomics has emerged as an important area for cancer research. Chromatin elements, such as promoters and enhancers, are found to vary dramatically in tumor cells. Enhancers locate not only close to transcription start sites (TSS), but also in regions faraway from TSS, often over several hundreds of kilo base away, so called distal enhancers [8–10]. Enhancers are usually marked with H3K4me1, catalyzed by histone methyltransferase *lysine methyltransferase 2* *C/D* (*KMT2C/D*, also named as *MLL3/4*) [11, 12]. The active enhancers are further labelled with H3K27ac and bound by mediator complex, which facilitates enhancer-promoter crosstalk [10, 13, 14]. Nowadays, H3K27ac is often used as a mark to identify active enhancers; and *E1A binding protein p300* (*EP300*, also known as *p300*) and *CREB binding protein* (*CEBBP*, also known as *CBP*) are the corresponding histone acetyltransferases [15, 16]. Enhancers within certain ranges are gathered together and those with highest MED1 or H3K27ac signals are called as super enhancers [17–20]. Super enhancers are believed to contain high transcription activity, and is essential for the expression of key genes controlling important phenotypes in the cell and useful for cell identification. In tumor cells, super enhancers are the potential critical regulatory elements on chromatin, often associated with activated oncogenes [18].

Gain of enhancer activity has been hypothesized as one of the common features for cancers [10, 18, 21, 22], which is supported by recent studies in patients and animal models [15, 23, 24]. However, it is still not clear whether it is a common feature for all the cancers or just a portion of them. Interestingly, many genes related with epigenetic regulation of enhancer activity are frequently mutated in cancer, such as *MLL3/4*, *p300*, *CBP*, *lysine demethylase 6A* (*KDM6A*, also named as *UTX*) and *lysine demethylase 5C* (*KDM5C*) [21, 25–29]. KDM5C, also known as JARID1C or SMCX, is a demethylase for H3K4me2/3 in mammalian cells [30]. It is located on enhancers and catalyzes H3K4me3 to H3K4me1 to keep the normal functions of enhancers [25, 31, 32]. If KDM5C is mutated or repressed, H3K4me3 will be enriched on enhancers and cause over activation of target genes [25, 33]. Recently, KDM5C was also reported to be able to promote gene expression through recruiting p300 to enhancer independent on its enzyme activity [34]. KDM5C is considered as a tumor suppressor through regulating enhancer function in several types of cancer, such as breast cancer, clear cell renal carcinoma and cervix cancer [25, 33, 35]. However, it is also reported to contain oncogenic functions in the cells of other types of cancer [34, 36]. Thus, the functions of KDM5C are quite controversial, and it is important to clarify the underlying mechanisms.

Ubiquitination dependent protein degradation is one of the important processes for cancer regulation. TRIM11 is one ubiquitin E3 ligase containing ring finger domain and recently reported to be involved in cancer [37–40]. TRIM11 increases proteasome activity through binding to proteasome and USP14 [41]. It has also been reported to promote lung adenocarcinoma via activation of STAT3/VEGFA signaling [42], and enhance lymphomas by targeting Axin1 and activating β-catenin signaling [43]. But all the above studies are completed in cell-based models, and the roles of TRIM11 in vivo have not been determined.

In the current study, we investigate the roles of TRIM11 in a genetic knockout animal model, and discover KDM5C as one substrate for TRIM11-mediated ubiquitination in vivo and in vitro. Our study demonstrates that TRIM11 promotes breast cancer through targeting KDM5C and reprogramming epigenetic modifications on enhancers.

## Materials and methods

### Reagents and cell lines

HEK-293T, HCT15, HepG2, DU145, UMUC3, HeLa, SW480, EJ, ZR-75-30 and HL7702 cell lines were cultured in Dulbecco’s Modified Eagle Medium (DMEM, Gibco) supplemented with 10% fetal bovine serum (FBS, BI), 1% penicillin and streptomycin at 37 °C with 5% CO2. 769-P, 786-O, PC3, SV-HUC-1, PNT1A OS-RC-2, Li-7, U2OS, and 22RV1 cell lines were cultured in RPMI 1640 (Gibco) supplemented with 10% FBS, 1% penicillin and streptomycin at 37 °C with 5% CO2. MDA-MB-231 and MDA-MB-468 cell lines were cultured in Leibovitz’s L-15 (Gibco) supplemented with 10% FBS, 1% penicillin and streptomycin at 37 °C with 100% air. ZR-75-30 was gifted from Dr. Fei Lan of College of Life Sciences, School of Basic Medical Sciences and Institutes of Biomedical Sciences, Fudan University, Shanghai 200032, China. The other cell lines were purchased from Cell Bank of Chinese Academy of Science.

Antibodies recongnizing Flag (Sigma F3165, RRID:AB_259529), HA (Origene TA100012; Abcam ab9110, RRID:AB_307019), Myc (Abclonal AE038), EGFP (Abmart 264076), β-Actin (Abclonal AC026, RRID:AB_2768234), GAPDH (Abclonal AC002, RRID:AB_2736879), H3 (Abcam ab1791, RRID:AB_302613), TRIM11(Abcam ab111694, RRID:AB_10900962; Sigma HPA028541, RRID:AB_10600833), KDM5C (Abcam ab34718, RRID:AB_881090; Bethyl A301-034A, RRID:AB_817995; Abclonal A9911, RRID:AB_2770027), MCAM(Abclonal A9703, RRID:AB_2863760), H3K27ac (Abcam ab4729, RRID:AB_2118291) and H3K4me3 (Millipore 04-745, RRID:AB_1163444; Abcam ab12209, RRID:AB_442957) were purchased from the indicated manufacturers. MG132, Chloroquine (CQ), ammonium chloride (NH4Cl) and anti-Flag M2 magnetic beads were purchased from Sigma-Aldrich. 3-Methyladenine(3-MA) was purchased from MCE. Protein G Sepharose beads were from GE Healthcare. PCR primers were custom synthesized by TSINGKE or Huayu Gene and siRNAs by GenePharma.

### Ethics approval and consent to participate

All the animal operations were following the laboratory animal guidelines of Wuhan University and were approved by the Animal Experimentations Ethics Committee of Wuhan University (Protocol NO. 14110B). The patient tissue chips were purchased from Avilabio. No patient study was involved and the consent to participate is not applicable.

### Mice model and animal housing

Trim11^+/−^ C57BL/6N were constructed by Beijing Biocytogen Co. Ltd. Mice were then mated with FVB/N-Tg (MMTV-PyVT) 634Mul/J transgenic mice (Model Animal Center, Nanjing University) after at least three generations of backcrossing. *Trim11*^*+/+*^ *MMTV-PyVT* and *Trim11*^*+/*−^ *MMTV-PyVT* female mice were identified. Mice were palpated twice weekly for the appearance of mammary tumors. Tumor latency was the age at which the first tumor was palpated. Palpable mammary tumors were measured every 4 days, and mice were sacrificed at the third month. Tumors were harvested, weighed and stored for further analysis. Tumor volumes were calculated as V = 0.5 × length × width^2^. Data were shown as mean ± SD or SEM and *p* value was calculated by the student’s *t* test.

All the mice were born and maintained under pathogen-free condition at 20–24 °C with a humidity of 40–70% and a 12/12 h dark/light cycle (lights on at 7:00 AM, lights off at 7:00 PM), with free access of water and food (Animal Center of College of Life Sciences, Wuhan University).

### Immunoblot analysis

Cell lysates were prepared with SDS lysis buffer (50 mM Tris-HCl pH 6.8, 4% SDS). Tissue lysates were prepared with strong RIPA buffer (50 mM Tris-HCl pH 7.4, 150 mM NaCl, 1% Triton X-100, 1% sodium deoxycholate, 0.5% SDS) and homogenized. Lysates were separated by SDS-PAGE and transferred to nitrocellulose filter membranes. After blocking, the blots were incubated with primary antibodies at 4 °C. Then the blots were washed three times in TBS-T, incubated with secondary antibodies at room temperature for 1 h, and detected by Clarity Western ECL Substrate (BIO-RAD).

### Immunoprecipitation

Cells were harvested and lysed in NP40 Lysis buffer (50 mM Tris, pH 7.4, 150 mM NaCl, 0.5% NP40) or high salt lysis buffer (20 mM HEPES pH 7.4, 10% glycerol, 0.35 M NaCl, 1 mM MgCl2, 0.5% Triton X-100, 1 mM DTT) in the presence of proteinase inhibitors. After removing insoluble particles, the supernatant was incubated with protein G beads (GE Healthcare) and specific antibodies at 4 °C for 4 h. The beads were spin down and washed three times with NP40 buffer. SDS loading buffer was then added to the beads for SDS-PAGE and western blotting.

### Reverse transcription and quantitative PCR

Total RNA from cultured cells was extracted with RNA extraction kit (Aidlab). For RNA extraction from tissues, 20 mg tissues were used and total RNA was extracted with RNA extraction kit (CWBIO). Approximately 1 μg of total RNA was used for reverse transcription with a first strand cDNA synthesis kit (Toyobo or Vazyme). The resulted cDNA was then assayed with quantitative PCR. β-actin was used for normalization. The sequences of primers are in Supplementary Table 11. Assays were repeated at least three times. Data were shown as average values ± SD or SEM. *P* value was calculated using student’s *t* test.

### Protein expression in bacteria and GST purification

TRIM11 isoformX2 cDNA sequences were cloned into pGEX-KG vector. The constructs were transformed into BL-21 bacteria, and induced with 1 mM IPTG at 18 °C for 4 h. The harvested cells were lysed by high pressure crusher and the lysates were centrifuged at 10,000 *g* for 1 h. Recombinant proteins were purified from the supernatant with Glutathione Sepharose 4 according to manufacturer’s instructions (GE Healthcare). Protein concentration was quantified by Qubit 2.0 (Invitrogen).

### In vitro ubiquitination assays

The plasmids and purified proteins of E1, E2 and Ub are gifts of Dr Wei Li from Institute of Zoology, CAS. GST-TRIM11 were expressed and purified from bacteria. The Flag-KDM5C was expressed in 293T and purified by Flag M2 magnetic beads. In vitro ubiquitination assays were carried out by adding 0.5 μg GST-UAE1, 1 μg His-UbcH5b, 1 μg Flag-KDM5C, 1 μg His-Ub and 5 μg GST-TRIM11 in ubiquitination buffer (20 mM Tris-HCl pH 7.4, 10 mM ATP,10 mM MgCl_2_, 0.1 mM DTT) to a final volume of 50 μl. The reactions were incubated at 37 °C for 1 h. The samples were diluted tenfold in lysis buffer (50 mM Tris-HCl pH 7.4, 150 mM NaCl, 0.5% NP-40, 1 mM Aprotinin, 1 mM Leupeptin) for immunoprecipitation or immunoblot analysis.

### Real-time cell analysis (RTCA) of cell proliferation

5000 per well of Cells were cultured in CIM-Plate wells. The cell index signals were read by xCELLigence RTCA DP Analyzer (ACEA bioscience Inc). Cell proliferation was monitored continuously over a 96 h period. Each experiment was repeated three times and results were presented as mean ± SD.

### MTT assay for cell proliferation

Cells were seeded on a 96-well plate and cultured for 24, 48, 72, or 96 h. 5 μl MTT (5 μg/μl) was added into each well, and incubated for 4 h at 37 °C. Four hundred microliters of lysate buffer (50% DMF + 30% SDS, pH 4.7) was added into each well followed by 4 h incubation at 37 °C. The absorbance at 570 nm was measured by Microplate System. Assays were repeated at least three times. Data were shown as mean ± SD and *p* value was calculated by the student’s *t* test.

### Transwell assay

4 × 10^4^ MDA-MB-231, 1 × 10^5^ MDA-MB-468 or 4 × 10^4^ ZR-75-30 cells were plated in medium without serum in the upper chamber with a Matrigel-coated membrane (24-well insert; pore size, 8 µm; BD Biosciences), and medium supplemented with 10% fetal bovine serum was used as a chemoattractant in the lower chamber. After 24 h of incubation, cells on the lower surface of the membrane were fixed with methanol and stained with crystal violet, then photographed under the microscope. Four visual fields were taken for each cell line and cells were counted with ImageJ. Assays were repeated at least three times. Data were shown as average values ± SD of representative experiments and *p* value was calculated using student’s *t* test.

### Wound healing assay

4 × 10^5^ cells were plated on 12-well plates for 24 h and wounded by scratching with a pipette tip. Subsequently, the cells were photographed under the microscope at 0, 12, 24 h. Then calculating the percentage of wound closure. Assays were repeated at least three times.

### Cell cycle analysis with flow cytometry

Cells were harvested after digestion with 0.05% Trypsin-EDTA, washed twice with PBS and fixed in ice-cold 70% ethanol overnight. Fixed cells were washed twice with PBS and stained in PBS containing propidium iodide (PI, 50 μg/mL) and RNase (100 μg/mL) for 30 min at 37 °C. Cell cycle analysis was performed on an Epics XL-MCL flow cytometer (Beck- man Coulter) with System II (version 3.0) software (Beckman Coulter).

### Xenograft experiments of cancer cells

Female BALB/cJGpt-Foxn1nu/Gpt nude mice were purchased from Jiangsu GemPharmatech. The mice were grouped randomly and 5 × 10^6^ MDA-MB-231 cells in 100 μl PBS with matrigel were injected in the flank regions. All mice were sacrificed 72 days after injection, and tumors were harvested and weighed. All animal xenograft experiments were performed following the university laboratory animal guidelines and were approved by the Animal Experimentations Ethics Committee of Wuhan University. Tumor volumes were derived as V = 0.5 × length × width^2^. Data were shown as mean ± SD or SEM and *p* value was calculated by the student’s *t* test.

### H&E staining and IHC analysis

Patient breast cancer tissue chips were purchased from Shanxi Avila Biotechnology Co. Ltd (Supplementary Table 12). For IHC, tissue sections were deparaffinized, rehydrated and treated for heat-mediated antigen retrieval. Next, the sections were incubated in 3% H_2_O_2_ for 15 min at room temperature to quench the activity of endogenous peroxidase. After incubation in 3% BSA for 30 min, the sections were treated with primary antibody for TRIM11 and KDM5C (dilution 1:50) at 4 °C overnight. Then the sections were washed three times with PBS and treated for 30 min with horseradish peroxidase labeled goat-anti-rabbit IgG. After washed four times with PBS, the slide was visualized by DBA developer (Servicebio, G1211), and nuclei were stained with haematoxylin before examination by Olympus BX51 microscope.

### Generation of knockout/knockdown cell line with CRISPR/Cas9 system

The small guide RNA (sgRNA) sequences were designed by using the CRISPR Design Tool (http://tools.genomeengineering.org), provided by Feng Zhang lab. The target sequences sgRNAs were shown in Supplementary Table 11. The oligo pairs containing the 20 nt guide sequences were annealed and ligated into the CRISPR plasmid (pLenti-v2-Cas9-gRNA). To construct knockdown cell lines, the lentiviral particles were produced in HEK-293T cells. Then the supernatant was used to infect the desired cells, which were selected by puromycin and validated by western blotting.

### ChIP assay

ChIP assay was performed as previously described [24]. Briefly, Cells were cross-linked with 1% formaldehyde for 10 min at room temperature and quenched with 0.125 M glycine for 5 min. Cross-linked cells were washed twice with PBS, then collected by centrifugation. Cells were treated with lysis buffer (50 mM Tris-HCl pH 8.0, 0.5% SDS, 5 mM EDTA), and DNA was sonicated to 250–500 bp. Supernatant was collected and equally divided after diluted with five times of sonication dilution buffer (20 mM Tris-HCl pH 8.0, 150 mM NaCl, 2 mM EDTA, 1% Triton X-100). The samples were then incubated with protein G beads and antibodies at 4 °C overnight. The beads were washed with wash buffer I, II, and III and then twice with TE. DNA was eluted with ChIP elution buffer (0.1 M NaHCO3, 1% SDS, 20 μg/ml proteinase K), incubated at 65 °C overnight and extracted with DNA purification kit (TIANGEN DP214-03). ChIP-qPCR assays were repeated at least three times. Data were shown as average values ± SD and *p* value was calculated using student’s *t* test. The sequences of primers are in Supplementary Table 11.

### ChIP-Seq and data analysis

ChIP-seq libraries were constructed using VATHS Universal DNA Library Prep Kit for Illumina (Vazyme ND606). Libraries were sequenced by Illumina Hiseq Xten platform with pair-end reads of 150 bp. For ChIP-Seq analysis, Fastqc (https://www.bioinformatics.babraham.ac.uk/projects/fastqc/) was used for raw data quality control. Cutadapt (https://cutadapt.readthedocs.io/en/stable/) was used to remove law quality bases and library adaptor contamination. After data filter, quality control of clean reads was performed by Fastqc again. Bowtie2(version 2.3.5.1) (http://sourceforge.net/projects/bowtie-bio/files/) was used for data mapping to human reference genome hg19. Samtools(version1.4.1) (http://www.htslib.org) was used to sort BAM file and filter duplicate reads. Only unique mapped reads were accepted for further analysis. MACS2 (version2.1.1.2) (https://pypi.org/project/MACS2/) was used for ChIP-Seq peaks calling with *p* value cut-off 1e−8. Then HOMER annotatePeaks.pl (https://www.biostars.org/p/9475396/) was used to annotate ChIP-seq peaks compare to reference genome hg19.

### RNA-seq and data analysis

RNA-seq libraries were constructed with NEBNext Poly(A) mRNA Magnetic Isolation Module (NEB E7490) and NEBNext Ultra II Non-Directional RNA Second Strand Synthesis Module (NEB E6111). RNA-seq libraries were sequenced by Illumina Nova-seq platform with pair-end reads of 150 bp. Quality control of mRNA-seq data was performed using Fatsqc (https://www.bioinformatics.babraham.ac.uk/projects/fastqc/) and low-quality bases were trimmed. The adaptor sequence was removed using Cutadapt (https://cutadapt.readthedocs.io/en/stable/) to clean RNA-seq raw data. All RNA-seq data were mapped to the human reference genome (hg19) or mouse reference genome (mm10) by HISAT2 (version 2.1.0) (http://daehwankimlab.github.io/hisat2/download/). The gene expression level was calculated by Cufflinks with default parameters. Differential expressed genes (DEGs) were identified by 1.5-fold change and *p* value < 0.05. Gene ontology analysis and KEGG pathways analysis was performed using DAVID (https://david.ncifcrf.gov).

### Bioinformatic analysis of clinical data

Overall survival analysis of gene expression and the correlation analysis in patient BRCA tumors were carried out via GEPIA platform (http://gepia.cancer-pku.cn/). The log-rank test was used for hypothesis evaluation. Values of *p* < 0.05 were considered as significantly different. Breast cancer gene expression microarray and corresponding clinical data used in this study were downloaded from publicly available GEO databases (http://www.ncbi.nlm.nih.gov/geo/).

### CRISPR-Cas9-KRAB-mediated enhancer repression

Site-specific single guide RNAs (sgRNAs) targeting distal enhancers were designed with a public filtering tool (https://zlab.bio/guide-design-resources) to minimize off-target cleavages. For CRISPR interference, sgRNAs were cloned into pLH-spsgRNA2 (Addgene, #64114) through a BbsI site according to the protocol recommended. Lentivirus was generated in HEK293T cells, and Stable cell lines were generated by infecting MDA-MB-231 with lentiviruses expressing dCas9-KRAB-MeCP2 and sgRNAs. Cells were then screened with puromycin (1 μg/ml, Amresco) and hygromycin (200 μg/ml, Roche) for 48 h, and examined by RT-qPCR.

### Statistics and reproducibility

For experiments other than NGS sequencing, at least three times for each experiment were performed with similar results and different biological replicates. Data are presented as mean values ± SD or SEM. Statistical analysis was performed using unpaired two-tailed Student’s *t* test. *p* value was either labelled on the corresponding items or listed in the legends. Statistical significance was assigned with *p value ≤ 0.05, **p value ≤ 0.01, *** *p* value ≤ 0.001.

## Results

### Exogenous expressed TRIM11 regulates KDM5C protein stability in mammalian cells

**Fig. 1:**
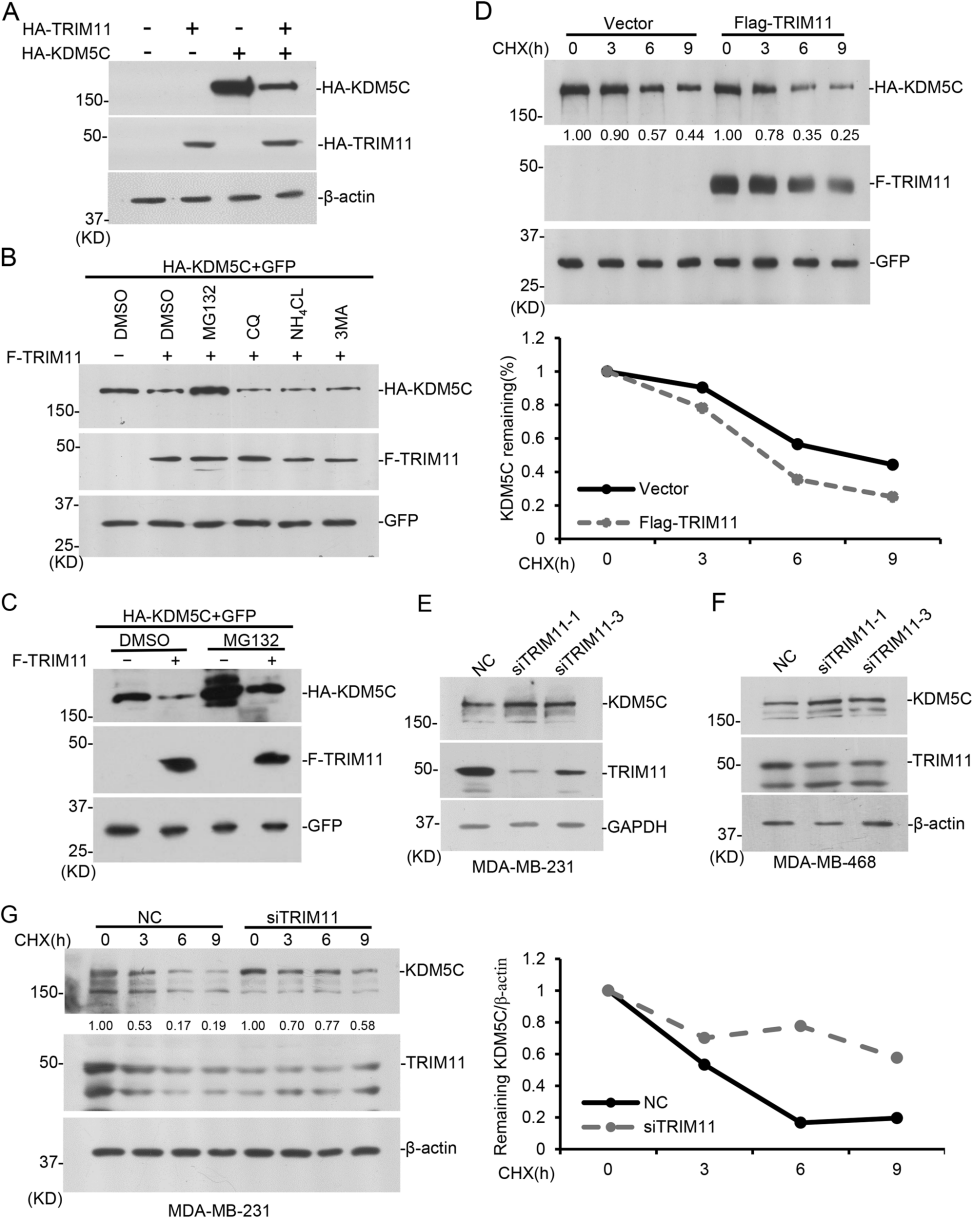
TRIM11 regulates KDM5C stability. **A** Lentiviruses containing phage-HA-TRIM11 or phage-HA-KDM5Cwere prepared and used to infect MDA-MB-231 cells. Stable cell lines were selected by puromycin. Western blotting was performed as indicated. **B** 293T cells were transfected with indicated plasmids. After 24 h, cells were treated with DMSO, MG132 (10 μM for 12 h), CQ (20 μM for 9 h), NH_4_Cl (20 mM for 9 h), 3-MA (5 mM for 9 h), and harvested for western blot. **C** 293T cells were transfected with indicated plasmids for 36 h followed by treatment with 10 μM MG132 for 12 h, and harvested for western blot. **D** 293T cells were transfected with indicated plasmids and treated with 100 μg/ml CHX for indicated time. Cells were harvested for western blot. HA-KDM5C abundance was quantified by ImageJ and plotted as indicated. **E**, **F** *TRIM11* was knocked down in MDA-MB-231 (**E**) or MDA-MB-468 (**F**) cells with siRNAs. After 48 h, cells were harvested for western blot as indicated. **G** *TRIM11* was knocked down by siRNA in MDA-MB-231 cells, and 48 h later cells were treated with 100 μg/ml CHX for indicated time. Cells were harvested for western blot. KDM5C abundance was quantified by ImageJ and plotted as indicated.

To investigate the potential regulator for histone demethylase KDM5C, we screened a pool of ubiquitin E3 ligases and identified TRIM11 as a candidate. We established stable expression cell lines of HA-KDM5C and HA-TRIM11 in MDA-MB-231 breast cancer cells, and verified the function of exogenous expressed TRIM11 on down-regulating KDM5C (Fig. 1A). To investigate whether TRIM11 regulates KDM5C through proteasome-mediated degradation, we treated cells with MG132 (proteasome inhibitor), chloroquine (CQ, autophagy inhibitor), NH_4_Cl (lysosome inhibitor) or 3MA (autophagy inhibitor). MG132, but not others, successfully rescued KDM5C protein level impaired by TRIM11 expression (Fig. 1B,C). To further confirm the role of TRIM11 on KDM5C, we expressed HA-tagged KDM5C with or without Flag-tagged TRIM11 and treated with cycloheximide (CHX). The results showed that KDM5C decayed faster in presence of exogenous expressed TRIM11 (Fig. 1D), indicating that TRIM11 regulates KDM5C protein stability.

### TRIM11 regulates KDM5C in breast cancer cells

To investigate whether endogenous TRIM11 regulates KDM5C, we knocked down *TRIM11* with different siRNAs in multiple cell lines, including breast cancer cell lines MDA-MB-231 and MDA-MB-468, kidney cell lines HEK293T, 769-P, 786-O and OS-RC-2, liver cell lines HepG2, HL7702 and Li-7, colon cell lines HCT15 and SW480, prostate cell lines PC3, DU145, 22RV1 and PNT1A, cervix cancer cell line HeLa, bladder cell lines EJ, SV-HUC-1 and UMUC3, and osteosarcoma cell line U2OS. We found that *TRIM11* knockdown elevated KDM5C protein level in breast cancer cell lines MDA-MB-231, MDA-MB-468, and kidney cell lines HEK293T, but did not affect its mRNA level (Fig. 1E, F, Supplementary Fig. S1B–E). The results suggest that TRIM11 selectively regulates KDM5C stability in breast cancer cells and HEK293T, but not other tested cell lines. We knocked down *TRIM11* in MDA-MB-231 and treated cells with CHX. The decay rate of endogenous KDM5C was lowered down with *TRIM11* deficiency (Fig. 1G). These indicate TRIM11 regulates the protein stability of endogenous KDM5C in certain cell lines.

### TRIM11 interacts with KDM5C

**Fig. 2:**
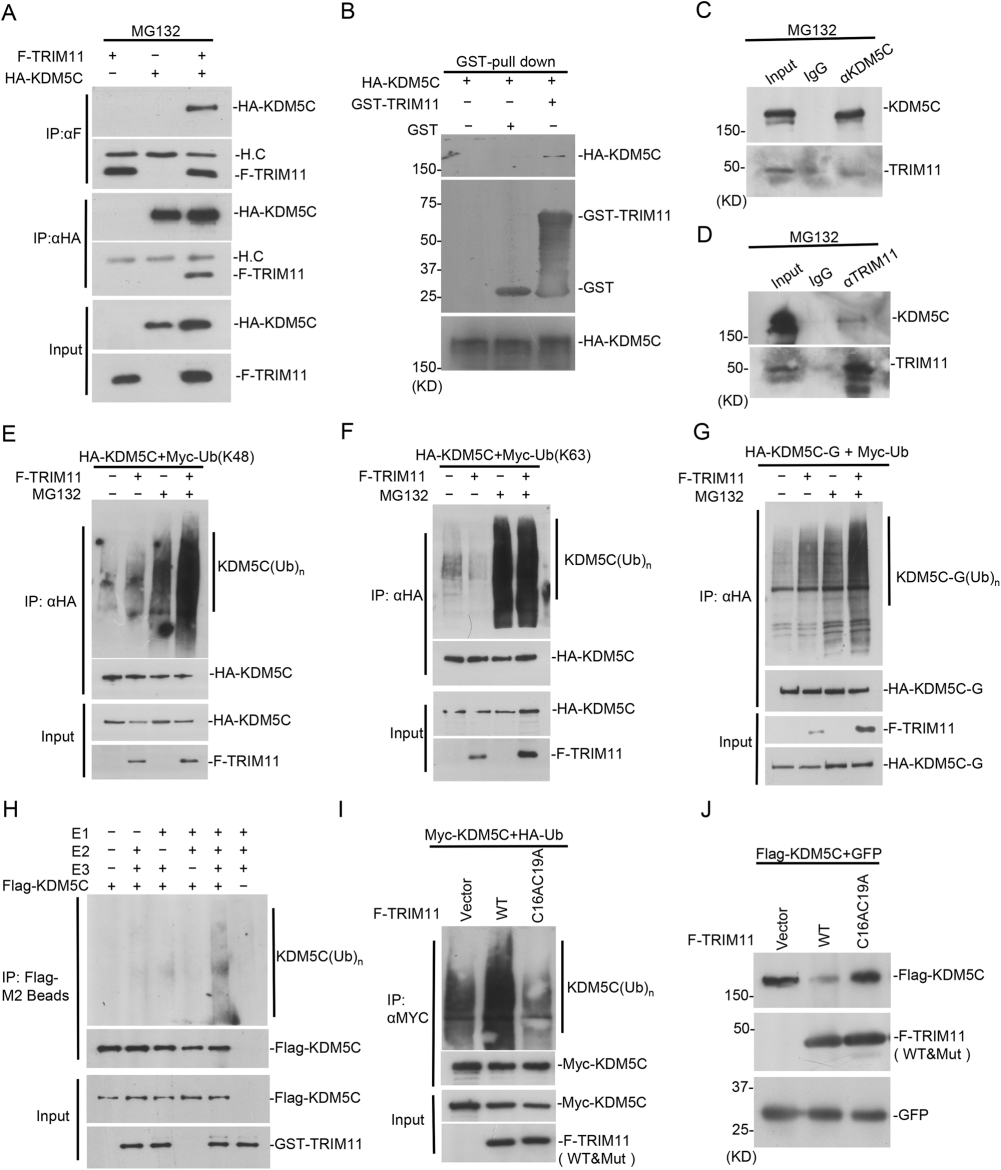
TRIM11 interacts with and poly-ubiquitinates KDM5C. **A** 293T cells were transfected with indicated plasmids for 36 h and treated with 10 μM MG132 for 12 h. Co-IP was performed with anti-Flag and anti-HA antibody. **B** GST-TRIM11 was expressed and purified from bacteria. HA-KDM5C was expressed in 293T cells and lysates were subject to GST pulldown assay. **C**,**D** MDA-MB-231 cells treated with MG132 were used for co-IP with KDM5C and TRIM11 antibodies, followed by western blot analysis. **E–G** 293T cells were transfected with HA-KDM5C (**E**, **F**)/HA-KDM5C-G (**G**), F-TRIM11 and Myc-Ub (WT/K48/K63) plasmids for 36 h followed by MG132 (10 μM) treatment for 12 h. Cell lysates were subjected to immunoprecipitation with anti-HA followed by western blot with anti-Myc. **H** In vitro ubiquitination assay was performed in the presence of Ub, E1, E2(UbCH5b), bacteria-purified GST-TRIM11 and 293T-purified Flag-KDM5C. **I** 293T cells were transfected with Myc-KDM5C, HA-Ub, F-TRIM11(WT) or F-TRIM11(C16AC19A). Ubiquitination of KDM5C was measured. **J** Flag-KDM5C, F-TRIM11(WT) and F-TRIM11(C16AC19A) were expressed in 293T cells and western blotting was performed as indicated.

To investigate whether TRIM11 interacts with KDM5C, we exogenously expressed Flag-TRIM11 with or without HA-KDM5C and treated cells with MG132. The result of co-immunoprecipitation showed that TRIM11 interacts with KDM5C (Fig. 2A). We then purified bacteria-expressed GST-TRIM11 and performed GST pulldown assay, which successfully pulled down HA-KDM5C from cell lysate (Fig. 2B). It indicates that TRIM11 probably directly interacts with KDM5C. Co-immunoprecipitation assays with antibodies recognizing KDM5C or TRIM11 indicate that endogenous TRIM11 also interacts with KDM5C (Fig. 2C, D). To map the domains of KDM5C responsible for TRIM11 interaction, a series of KDM5C truncations were constructed (Supplementary Fig. S2A). The results of co-immunoprecipitation assays show that KDM5C fragment A, C, D, F, G interacted with TRIM11, which suggest that the fragment (67-372AA) containing ARID and PHD domains is required for TRIM11 interaction (Supplementary Fig. S2A–G).

### TRIM11 is a ubiquitin E3 ligase for KDM5C

To study whether TRIM11 is a ubiquitin E3 ligase for KDM5C, we expressed HA-KDM5C and Myc-Ubiquitin (K48, all lysine mutated except K48) in HEK293T cells with or without TRIM11. TRIM11 expression increased the poly-ubiquitination chain on KDM5C and MG132 treatment further elevated KDM5C poly-ubiquitination level (Fig. 2E). TRIM11 exogenous expression also promoted the poly-ubiquitination of endogenous KDM5C in MDA-MB-231 (Supplementary Fig. S2I). As comparison, TRIM11 did not increase KDM5C ubiquitination level with Myc-Ubiquitin (K63, all lysine mutated except K63), indicating TRIM11 specifically promotes K48-linked poly-ubiquitination on KDM5C (Fig. 2F). To study the KDM5C fragments responsible for ubiquitination, we checked the poly-ubiquitination effect of the above fragments by TRIM11 and found that TRIM11 ubiquitinated KDM5C-G but not others (Fig. 2G, Supplementary Fig. S2B and H). We further performed in vitro ubiquitination assay with TRIM11 purified from bacteria and Flag-KDM5C purified from HEK293T, and the results show that TRIM11 is able to poly-ubiquitinated KDM5C in vitro (Fig. 2H). We further mutated two cysteine residues (C16 and C19) in the ring finger domain of TRIM11 to alanine and the results of ubiquitination assay show that the TRIM11 mutant could not catalyze KDM5C poly-ubiquitination and decrease KDM5C level, indicating the ubiquitin E3 ligase activity of TRIM11 is dependent on its ring finger domain (Fig. 2I, J). To examine the subcellular localization of TRIM11 and KDM5C, confocal analysis was performed with their antibodies, which showed that the endogenous TRIM11 and KDM5C are both localized in nuclear and cytosol (Supplementary Fig. S2J). All these together indicate that TRIM11 is a ubiquitin E3 ligase for KDM5C.

### TRIM11 regulates cancer cell proliferation and migration through KDM5C

**Fig. 3:**
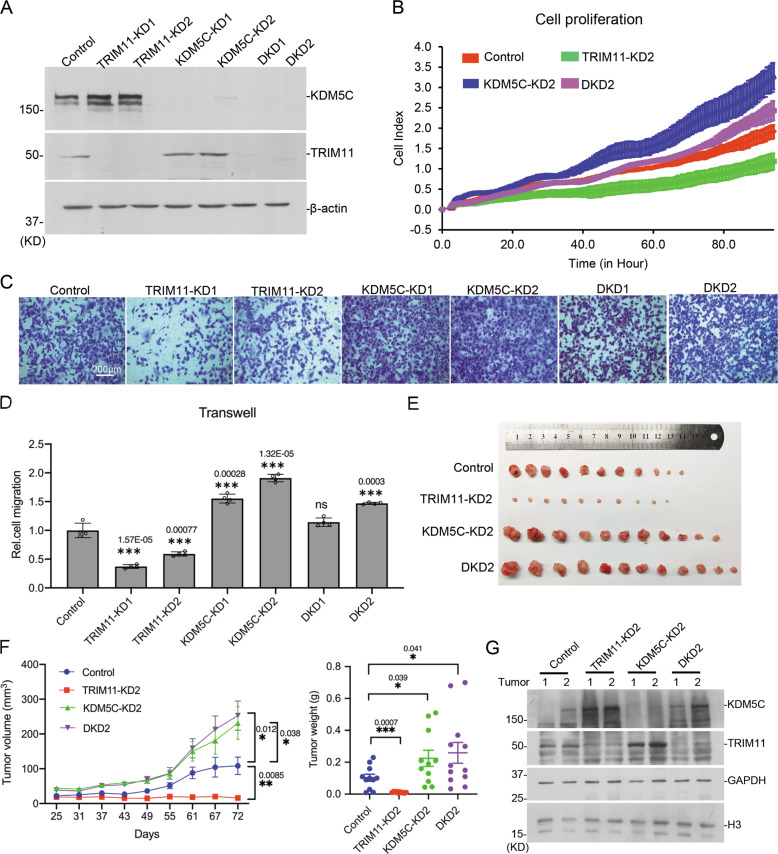
TRIM11 promotes proliferation and migration of breast cancer cells via degrading KDM5C. **A** TRIM11 knockdown (TRIM11-KD), KDM5C knockdown (KDM5C-KD) and Double knockdown (DKD) MDA-MB-231 cell lines were constructed using CRISPR/Cas9 system. Two sgRNAs were designed per gene. **B** Cell proliferation of the cells in (**A**) were monitored with real-time cell analysis (RTCA). **C**,**D** Cell migration of the above cell lines were tested by transwell assay. Four views of the cell migration images were taken for each cell line. The number of moving cells was counted by image J. Bar = 200 μm. **E** The indicated stable cells derived from MDA-MB-231 were injected into nude mice. The tumors were dissected and pictured. **F** Tumor growth curve (left) and tumor weight (right) were shown as mean ± SEM. ^*^p value ≤ 0.05, ^**^p value ≤ 0.01, ^***^p value ≤ 0.001. **G** Two tumors from each group was random picked and assayed with western blotting to confirm they are from original cell lines.

To investigate the roles of TRIM11 and KDM5C in cancer cells, we first constructed stable expressed cell lines of TRIM11 and KDM5C in MDA-MB-231. Cell proliferation assay with MTT showed that TRIM11 expression enhanced proliferation significantly, and KDM5C expression repressed cell proliferation (Supplementary Fig. S3A). Transwell assay showed that TRIM11 expression increased cell migration and KDM5C expression repressed migration enhanced by TRIM11 (Supplementary Fig. S3B,C). Flow cytometry results showed that TRIM11 expression increased the percentage of S phase and decreased the percentage of G1 phase (Supplementary Fig. S3D). Besides the above, we constructed stable cell lines of *TRIM11* knockdown, *KDM5C* knockdown and double knockdown (DKD) in MDA-MB-231 (Fig. 3A). Real time cell proliferation assay showed that *TRIM11* knockdown decreased cell proliferation, while *KDM5C* knockdown increased proliferation and rescued the phenotype caused by *TRIM11* knockdown (Fig. 3B). Transwell assay showed that *TRIM11* knockdown decreased cell migration rate, and *KDM5C* knockdown increased cell migration and rescued the phenotype caused by *TRIM11* knockdown (Fig. 3C, D). Scratch assay for cell migration supported the same conclusion (Supplementary Fig. S3E). *TRIM11* and *KDM5C* stable knockdown cell lines were also established in MDA-MB-468 and ZR-75-30 breast cancer cells. In MDA-MB-468-derived cell lines, their proliferation rates did not show significant difference, while *KDM5C* knockdown increased cell migration and *TRIM11* knockdown decreased it (Supplementary Fig. S4A–C). In ZR-75-30-derived cell lines, cell proliferation had no significant difference either, and *KDM5C* knockdown increased cell migration. Interestingly, *TRIM11* knockdown did not affect KDM5C level and did not significantly affect cell migration (Supplementary Fig. S4D–F). The results further support that TRIM11 regulates cell migration through KDM5C. These results together indicate that TRIM11 promotes breast cancer cell proliferation and migration. MDA-MB-468 is considered as a triple-negative breast cancer (TNBC) cell line, and ZR-75-30 for luminal B subtype. Our data suggest the regulation of KDM5C by TRIM11 is potentially specific for TNBC cells.

To investigate the roles of TRIM11 and KDM5C in breast cancer, we first injected the above stable knockdown cells derived from MDA-MB-231 into nude mice. The results showed that *TRIM11* knockdown greatly reduced the volume and weight of tumors, and *KDM5C* knockdown increased tumor growth (Fig. 3E–G). The DKD cells had no significant difference with *KDM5C* knockdown cells (Fig. 3E–G). It is probably because KDM5C acts downstream of TRIM11 and probably has an overwhelming effect on *TRIM11* knockdown.

### TRIM11 promotes breast cancer and regulates KDM5C in vivo

**Fig. 4:**
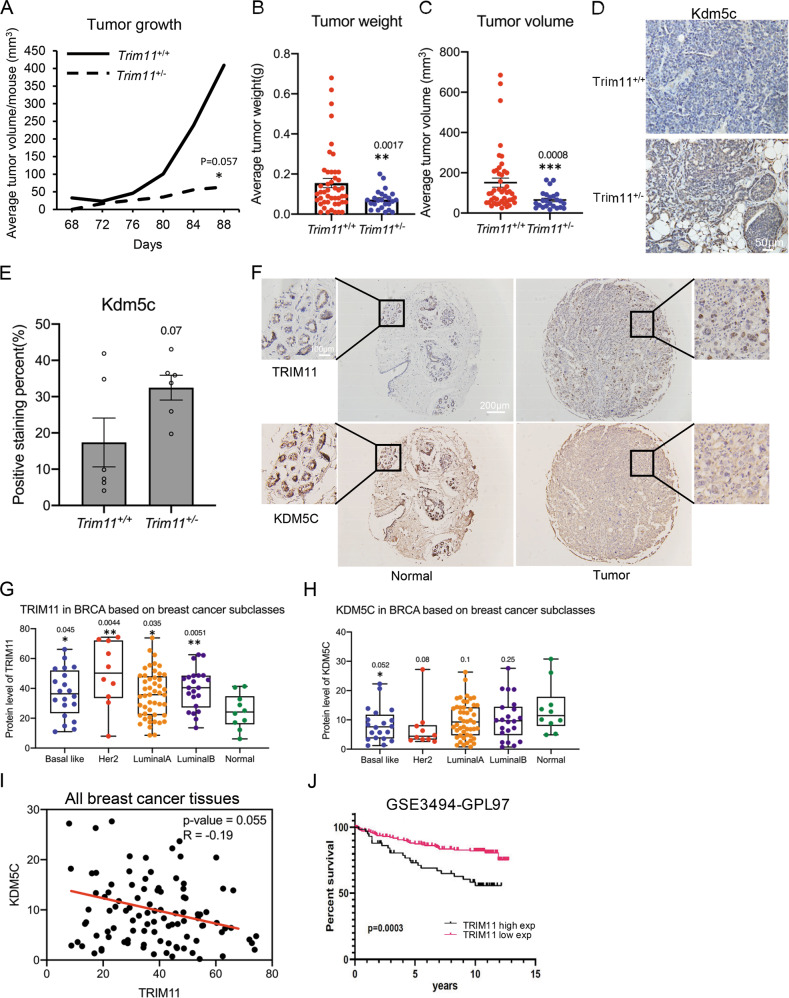
TRIM11 promotes mammary tumorigenesis and regulates KDM5C in vivo. **A** Tumor growth of Trim11 wild type or KO MMTV-PyVT mice, indicated as total tumor volume / mouse number (Trim11^+/+^ mice, *n* = 12; Trim11^+/−^ mice, *n* = 7). **B**, **C** The average weight and volume of breast tumors when sacrificed, shown as mean ± SEM (Trim11^+/+^ tumors, *n* = 46; Trim11^+/−^ tumors, *n* = 24). **D**, **E** Representative Kdm5c immunohistochemical (IHC) staining of breast cancer tissues from *Trim11*^+/+^ and *Trim11*^+/−^ *MMTV-PyVT* mice (*n* = 6). **F** Representative TRIM11 and KDM5C IHC staining in tumor tissue microarray (TMA). **G**, **H** Expression of TRIM11 and KDM5C in human breast cancer TMA was shown as mean, min to max and all points according to molecular subclasses. **I** Correlation of TRIM11 and KDM5C expression level in TMA. **J** Overall survival of BRCA patients with high and low TRIM11 expression was analyzed and plotted using GSE3494 datasets and the Kaplan–Meier method. Statistical analysis was performed using unpaired two-tailed Student’s *t* test (**A**, **B**, **C**, **D**, **G**, **H**), ^*^p value ≤ 0.05, ^**^p value ≤ 0.01, ^***^p value ≤ 0.001.

To further examine the role of TRIM11 in vivo, we constructed *TRIM11* knockout mouse model (Supplementary Fig. S5A, B). *TRIM11* knockout did not affect the growth and body weight obviously (Supplementary Fig. S5C). We crossed the mice with *MMTV-PyVT* breast cancer mouse model. Our results showed that the tumors of *Trim11*^+/−^*MMTV-PyVT* mice grew much slower compared with that of *Trim11*^+/+^*MMTV-PyVT* mice (Fig. 4A). Both tumor weight and volume were smaller in *Trim11*^+/−^*MMTV-PyVT* mice (Fig. 4B, C), supporting *TRIM11* as an oncogene (Fig. 4A–C). *TRIM11* deficiency did not affect *KDM5C* mRNA level in tumors (Supplementary Fig. S5D, E). Consistent with the above biochemical results, *TRIM11* knockout increased KDM5C level in tumor tissues (Fig. 4D, E, Supplementary Fig. S5F). RNA-Seq analysis was performed and the results suggested that in comparison with the tumors of wild type mice, the different expressed genes (DEGs) in the tumors of *TRIM11* deficiency mice were enriched in pathways of cell migration, immune response and metabolisms (Supplementary Fig. S5G, H, Supplementary Tables 1–3). All these indicated that *TRIM11* plays an oncogenic role in breast cancer animal model and regulates KDM5C in breast cancer tissues.

### Correlation of TRIM11 and KDM5C in breast cancer patients

To further explore the relationship between TRIM11 and KDM5C in breast cancer patient tissues, we performed immunohistochemical (IHC) staining with commercially available breast cancer tissue arrays (Fig. 4F). The result showed that TRIM11 mainly increased in nuclei, and KDM5C was high in the nuclei of normal tissue cells and low in the nuclei of tumor tissue cells (Fig. 4F). These suggest that in breast cancer tissues, TRIM11 probably mainly regulates KDM5C in nuclei. We divided the tissues into four subgroups according to the provided information of molecular marks, and found that TRIM11 was higher expressed and KDM5C was lower expressed in all four groups of breast cancer tissues (Fig. 4G, H). TRIM11 expression was correlated with progressive cancer stage, higher in all IIA, IIB, IIIA and IIIB stages, while KDM5C behaved the opposite (Supplementary Fig. S6A, B). TRIM11 was also expressed higher in high metastatic tumors and KDM5C was lower expressed (Supplementary Fig. S6C, D). The expression of TRIM11 and KDM5C in all tested tumor tissues was correlated, and the p value was very close to the significant value (*p* = 0.055) (Fig. 4I). For cancer subgroups, TRIM11 and KDM5C were significant correlated in Her2 subgroup (Supplementary Fig. S6E–H). Our IHC result gave us positive signals both in cytoplasm and nuclei, and TRIM11 probably only regulates nuclear but not cytoplasmic KDM5C (Fig. 4F), which may be the reason that the *p* value in the correlation analysis did not reach the significant value. Analysis with public data showed that *TRIM11* was highly expressed in breast cancer, as well as in many other types of cancer, compared with their corresponding normal tissues (Supplementary Fig. S6I, J), and the patients with higher *TRIM11* expression is associated with worse survival rates (Fig. 4J). The mRNA level of *KDM5C* is significantly lower in some breast cancer datasets (Supplementary Fig. S6K). These suggest that *TRIM11* plays oncogenic roles in many types of cancer, while *KDM5C* may be behaves as a tumor suppressor only in breast cancer.

### TRIM11 regulates H3K4me3 on enhancers through KDM5C

**Fig. 5:**
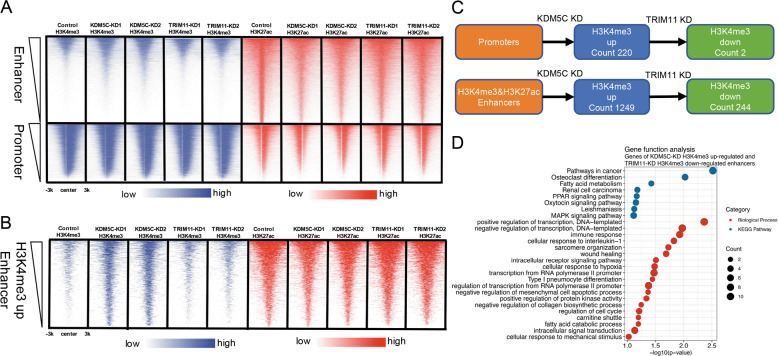
TRIM11 regulates H3K4me3 on enhancers through KDM5C. **A**, **B** Heat maps generated from ChIP-seq data showing the occupancy of H3K27ac and H3K4me3 on enhancers, promoters and KDM5C regulated enhancers in TRIM11-KD and KDM5C-KD MDA-MB-231 cell lines. **C** A sketch map to show the pipeline to identify promoters and H3K4me3-enriched enhancers regulated by TRIM11 and KDM5C. Differential H3K4me3 peaks in KDM5C-KD were identified by 1.5-fold change, and 1-fold change in TRIM11-KD. **D** Biological process and KEGG pathway enrichment analyses of adjacent genes controlled by enhancers whose H3K4me3 was up-regulated in KDM5C-KD and down-regulated in TRIM11-KD.

To further investigate the molecular mechanisms for TRIM11 and KDM5C in regulating breast cancer, we performed RNA-Seq and ChIP-Seq analysis in *TRIM11* or *KDM5C* knockdown cells derived from MDA-MB-231 (Supplementary Table 4). The analysis showed that in *KDM5C* knockdown cells the down-regulated DEGs were enriched in immune pathways (Supplementary Fig. S7A, Supplementary Table 5), and the up-regulated DEGs were enriched in pathways involved in immune response, metabolism and cell migration (Supplementary Fig. S7B, Supplementary Table 6). In *TRIM11* knockdown cells the down-regulated DEGs were enriched in Notch pathway and transcription process (Supplementary Fig. S7C, Supplementary Table 7), and the up-regulated DEGs were enriched in pathways involved in immune response, metabolism and cell migration (Supplementary Fig. S7D, Supplementary Table 8). *KDM5C* knockdown caused H3K4me3 upregulation on enhancers and *TRIM11* knockdown did not affect the global H3K4me3 obviously (Fig. 5A, B). However, when comparing promoters and H3K4me3-enriched enhancers, *TRIM11* knockdown affected much more H3K4me3 peaks on enhancers than promoters (Fig. 5C). The adjacent genes to the enhancers regulated by KDM5C and TRIM11 were enriched in pathway in cancer, immune response, fatty acid metabolism and wound healing (Fig. 5D). These suggest that TRIM11 regulates H3K4me3 on enhancers of cancer-related genes through targeting KDM5C.

### TRIM11 and KDM5C control *MCAM* expression through enhancer regulation

**Fig. 6:**
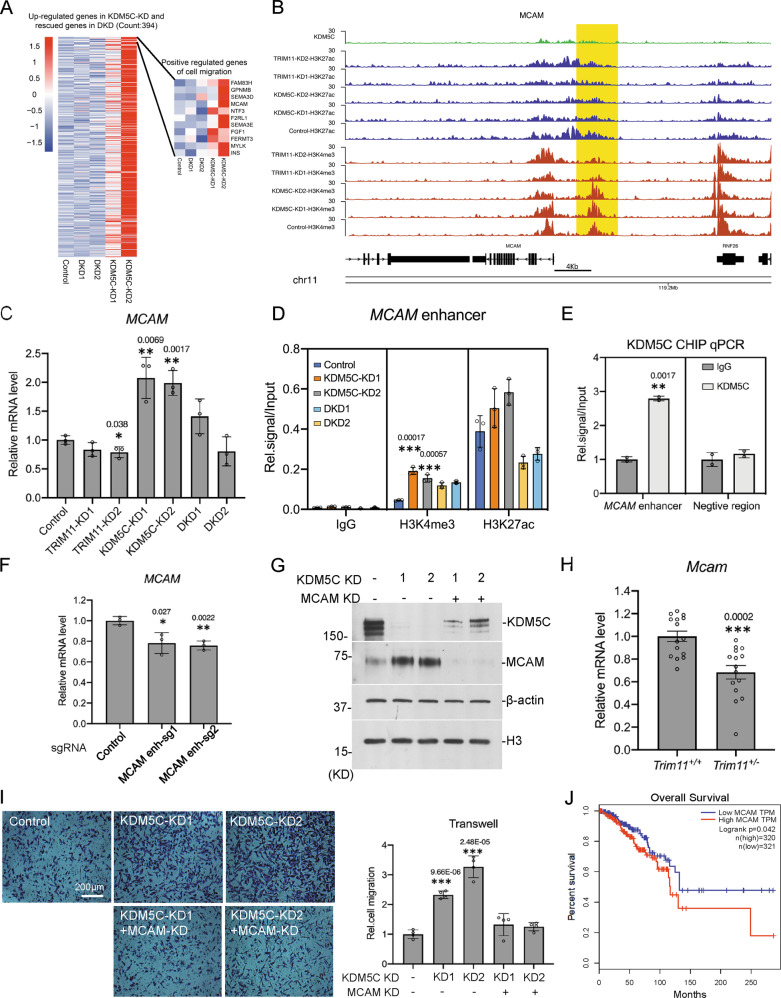
TRIM11 and KDM5C regulate cell migration through MCAM enhancer. **A** Heatmap of genes up-regulated in KDM5C-KD cell lines and rescued in DKD cell lines. Genes related to cell migration are highlighted at right. **B** The UCSC browser view to show KDM5C, H3K27ac and H3K4me3 enrichment on *MCAM* enhancer. **C** *MCAM* mRNA expression level in TRIM11-KD, KDM5C-KD and DKD cell lines (*n* = 3). **D** H3K27ac and H3K4me3 enrichment on *MCAM* enhancer in KDM5C-KD and DKD cell lines, analyzed by ChIP-qPCR (*n* = 3). **E** ChIP-qPCR analysis of KDM5C occupancy on the *MCAM* enhancer. IgG was used as the control. Statistics were presented as mean ± SD**. F** CRISPR-Cas9-KRAB system and two different sgRNAs were used and stable cell lines were established in MDA-MB-231. MCAM expression was detected with quantitative RT-PCR. **G** MCAM was stably knocked down in KDM5C knockdown MDA-MB-231 cells using the CRISPR/Cas9 system. **H** Cell migration of the cell lines in (**G**) were tested by transwell assay. Four views of the cell migration images were taken for each cell line. The number of moving cells was counted by image J. Relative cell migration was shown in the figure. **I** Quantitative RT-PCR analysis of *Mcam* gene in breast tumors of *Trim11*^+/+^ and *Trim11*^+/−^ MMTV-PyVT mice (*n* = 5, per group, 3 replicates). **J** Overall survival was analyzed and plotted using the Kaplan–Meier method. The survival rates for patients with high and low *MCAM* expression were plotted as red and blue lines, respectively. The number of patients in each group was shown in parentheses, *p* values were calculated using a log-rank test. Histograms were presented as mean ± SD (**C**, **D**, **E**, **G**) or SEM(**H**). ^*^p value ≤ 0.05, ^**^p value ≤ 0.01, ^***^p value ≤ 0.001.

KDM5C is a regulator of enhancer activity and its deficiency should upregulate the expression of its target genes. Since previous results showed that TRIM11 and KDM5C regulate cell migration, we examined the migration-related DEGs upregulated in *KDM5C* knockdown cells and rescued in DKD cells, to identify the potential target genes of TRIM11 and KDM5C (Fig. 6A, Supplementary Fig. S8A–C, Supplementary Table 9). ChIP-Seq analysis in *TRIM11* and *KDM5C* knockdown cells showed that one enhancer around 4 kb upstream of *MCAM* TSS had strong H3K4me3 signal, which increased after *KDM5C* knockdown and decreased in *TRIM11* knockdown cells (Fig. 6B). The mRNA level of *MCAM* increased in *KDM5C* knockdown cells and rescued in DKD cells (Fig. 6C). A previous published result of KDM5C ChIP-Seq analysis showed positive KDM5C peaks around the enhancer site (Fig. 6B) [25]. ChIP analysis with H3K4me3 and H3K27ac antibodies confirmed the change of histone modifications on the enhancer (Fig. 6D). ChIP assay showed that KDM5C was bound to *MCAM* enhancer site, which was confirmed with a HA-tagged KDM5C stable cell line (Fig. 6E & Supplementary Fig. S8D). A CRISPR-Cas9-KRAB system was used to study whether *MCAM* is the target gene of the identified enhancer [44]. Two different sgRNAs were designed and both of them repressed *MCAM* expression, supporting that the identified KDM5C-bound enhancer regulates *MCAM* (Fig. 6F). To examine the role of MCAM in cell migration, we knocked down *MCAM* in *KDM5C* knockdown MDA-MB-231 cells, and performed transwell and wound healing assays (Fig. 6G & Supplementary Fig. S8E). The result showed that *MCAM* knockdown suppressed the migration enhanced by *KDM5C* knockdown (Fig. 6H). In the breast cancer tissues of *Trim11*^*+/+*^ and *Trim*^*+/−*^ mice, *MCAM* expression was down-regulated with *Trim11* deficiency (Fig. 6I). All these indicate that TRIM11 and KDM5C regulate *MCAM* expression through targeting its enhancer. Analysis of patient survival rate with TCGA breast cancer data show that patients with higher *MCAM* expression have worse survival rate (Fig. 6J), further supporting that regulation of *MCAM* by TRIM11 and KDM5C is important for breast cancer.

### TRIM11 regulates inflammatory gene expression through KDM5C

The above RNA-Seq results found that many DEGs regulated by TRIM11 were enriched in immune response pathways (Supplementary Fig. S5F, G, S7C, D). A previous study has shown that TRIM11 regulates innate immune pathway through targeting TBK1, which is consistent with our results. However, we found that the DEGs which were down-regulated in *KDM5C* knockdown cells and rescued in DKD cells, were also enriched with immune response pathways (Supplementary Fig. S9A), suggesting KDM5C is also involved in the regulation of immune gene expression. We then confirmed the results in cells with quantitative RT-PCR, which showed that *KDM5C* knockdown decreased the expression of *IL1A*, *IL6*, *CXCL1*, *CXCL2*, *CXCL3* and *CXCL8*, while *TRIM11* knockdown increased their expression (Supplementary Fig. S9B, C). In the mouse tumor tissues, Trim11 deficiency also upregulates the expression of *Cxcl1*, *Cxcl2* and *Cxcl3* (Supplementary Fig. S9D). These suggest TRIM11 regulates immune gene expression through targeting KDM5C, which may serve as a new mechanism for TRIM11’s function in cancer immune response.

## Discussion

TRIM11 has been reported as one oncogene recently, but its in vivo functions have not been determined. KDM5C is one important tumor-suppressive regulator for oncogenic enhancers, but its regulatory mechanisms still remain elusive. Our study identified TRIM11 as a ubiquitin E3 ligase for KDM5C. TRIM11 interacts with KDM5C, catalyzes K48-linked ubiquitin chain on KDM5C and regulates KDM5C stability in breast cancer cells. TRIM11 regulates proliferation and migration of breast cancer cells through KDM5C, and TRIM11 promotes breast cancer and regulates KDM5C stability in an in vivo animal model. These data connect the oncogenic function of TRIM11 and tumor-suppressive function of KDM5C in breast cancer, which establish a new mechanism for breast cancer study.

KDM5C is an important regulator for enhancer function. We showed that TRIM11 regulates H3K4me3 on enhancers and gene transcription through KDM5C. In breast cancer, TRIM11 is involved in the regulation of multiple processes, including immune response, migration-related pathway and lipid metabolism. It has been reported that TRIM11 regulates innate immunity pathway through TBK1. Our data show that KDM5C is also involved in the regulation of inflammation genes, which suggest that TRIM11-mediated KDM5C degradation is probably also involved in the immune response in breast cancer. Enhanced cell migration is one important aspect for tumor cells. Our data show that TRIM11 and KDM5C regulate the expression of *MCAM* in breast cancer cells, through targeting an enhancer close to *MCAM* TSS. The detailed mechanisms how KDM5C is involved in breast cancer should be further investigated in the future.

Our IHC study of breast cancer patient tissues show that in breast cancers, TRIM11 is mainly located in nuclei. KDM5C is mainly located in the nuclei of normal tissue cells, and is much lower in the nuclei of tumor cells. It is probably that TRIM11 degrades KDM5C in the nuclei of tumor cells. We also observed relatively high KDM5C signal in the cytoplasm of tumor cells. It is possible that the KDM5C antibody recognizes unspecific signal, or TRIM11 does not regulate KDM5C in the cytoplasm.

To sum up, our study identified TRIM11 as a new ubiquitin E3 ligase for KDM5C in vivo and in vitro, and revealed new mechanisms for oncogenic enhancer regulation in breast cancer.

## Supplementary information

Sup Figures

Sup. Datasets

Original Data File

aj-checklist

## Data Availability

All the deep sequencing data were available at the GEO database with the accession number GSE185836. Breast cancer gene expression microarray and corresponding clinical data used in this study were downloaded from GEO database (http://www.ncbi.nlm.nih.gov/geo/): GSE3494 (based on GPL97 platform) and GSE10780. ChIP-Seq data for KDM5C were obtained from publicly available datasets in ArrayExpress (GSE71327), performed in ZR-75–30 cells.
