# Supplementary material for: Regulation of KDM5C stability and enhancer reprogramming in breast cancer
Source: Cell Death Dis. 2022 Oct 3;13(10):843. doi: 10.1038/s41419-022-05296-5 (PMC9530161; doi:10.1038/s41419-022-05296-5)
Supplement: Supplementary file 1 — Sup Figures [file 41419_2022_5296_MOESM1_ESM.pdf]

Supplemental Fig. S1

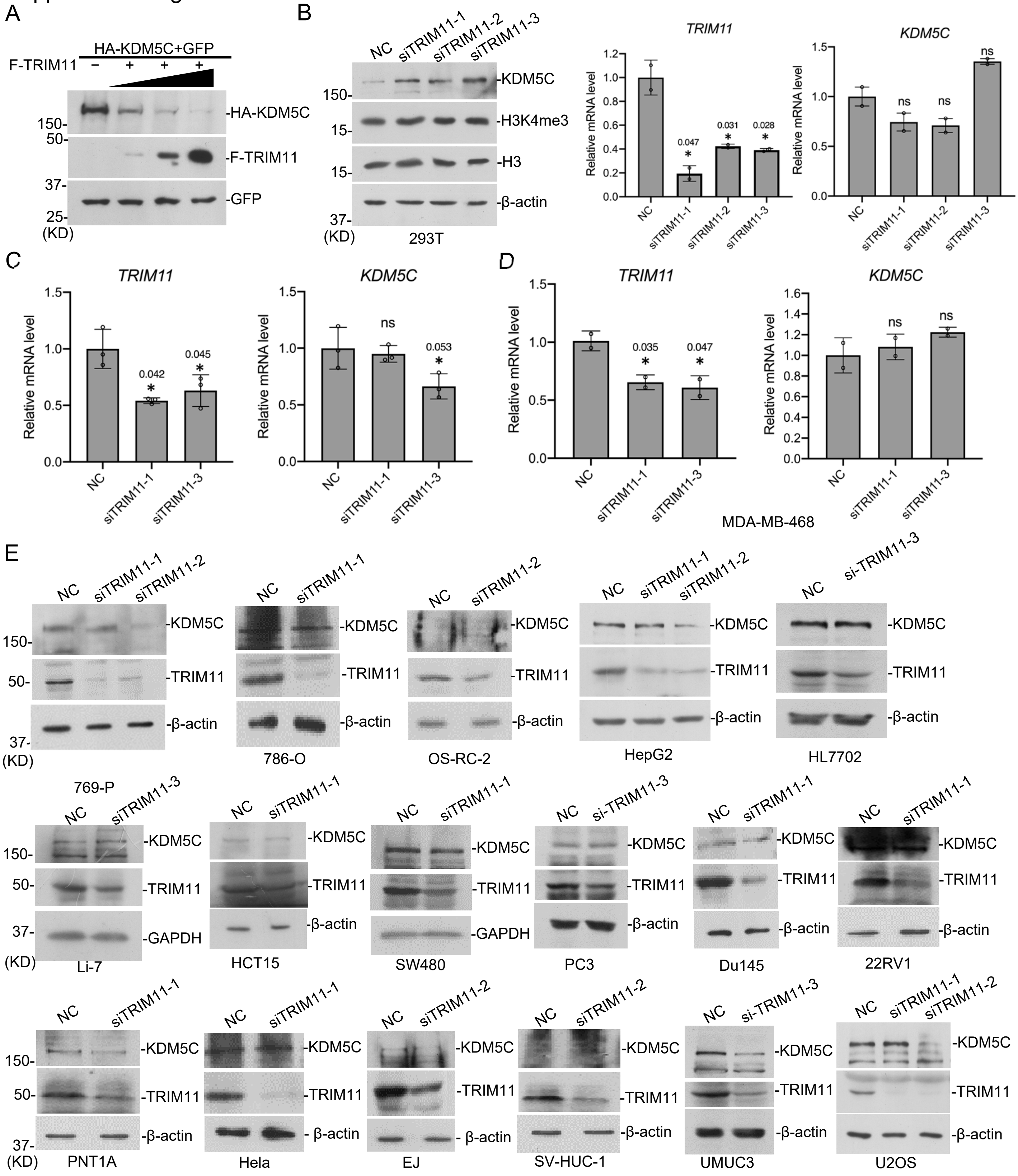

Sup. Figure S1 TRIM11 regulates KDM5C stability in HEK293T and breast cancer cells. (A) TRIM11 was overexpressed with a gradient in 293T to test the changes of KDM5C by western blot. **(B)** 293T were transfected with negative control or *TRIM11*-specific siRNAs. After 48 h, cells were harvested for western blot and Quantitative RT-PCR. **(C&D)** MDA-MB-231 and MDA-MB-468 were transfected with negative control or *TRIM11*-specific siRNAs. After 48 h, cells were harvested for quantitative RT-PCR analysis of *TRIM11* and *KDM5C* genes. **(E)** The indicated cell lines were transfected with control or *TRIM11*-specific siRNAs. After 48 h, cells were harvested for western blot. Histograms were presented as mean ± SD. * p-value < =0.05, ** p-value < =0.01, *** p-value < =0.001.

Supplemental Fig. S2

A

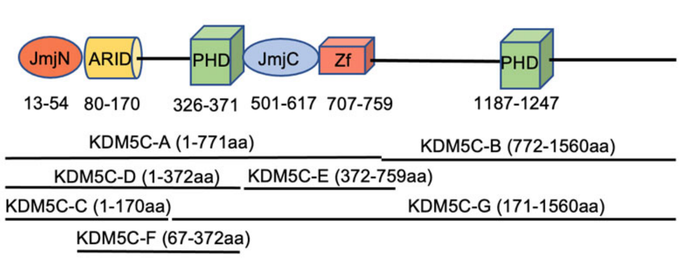

B

	Interaction with TRIM11	ubiquitination
KDM5C-A	+	–
KDM5C-B	–	–
KDM5C-C	+	–
KDM5C-D	+	–
KDM5C-E	–	–
KDM5C-F	+	–
KDM5C-G	+	+

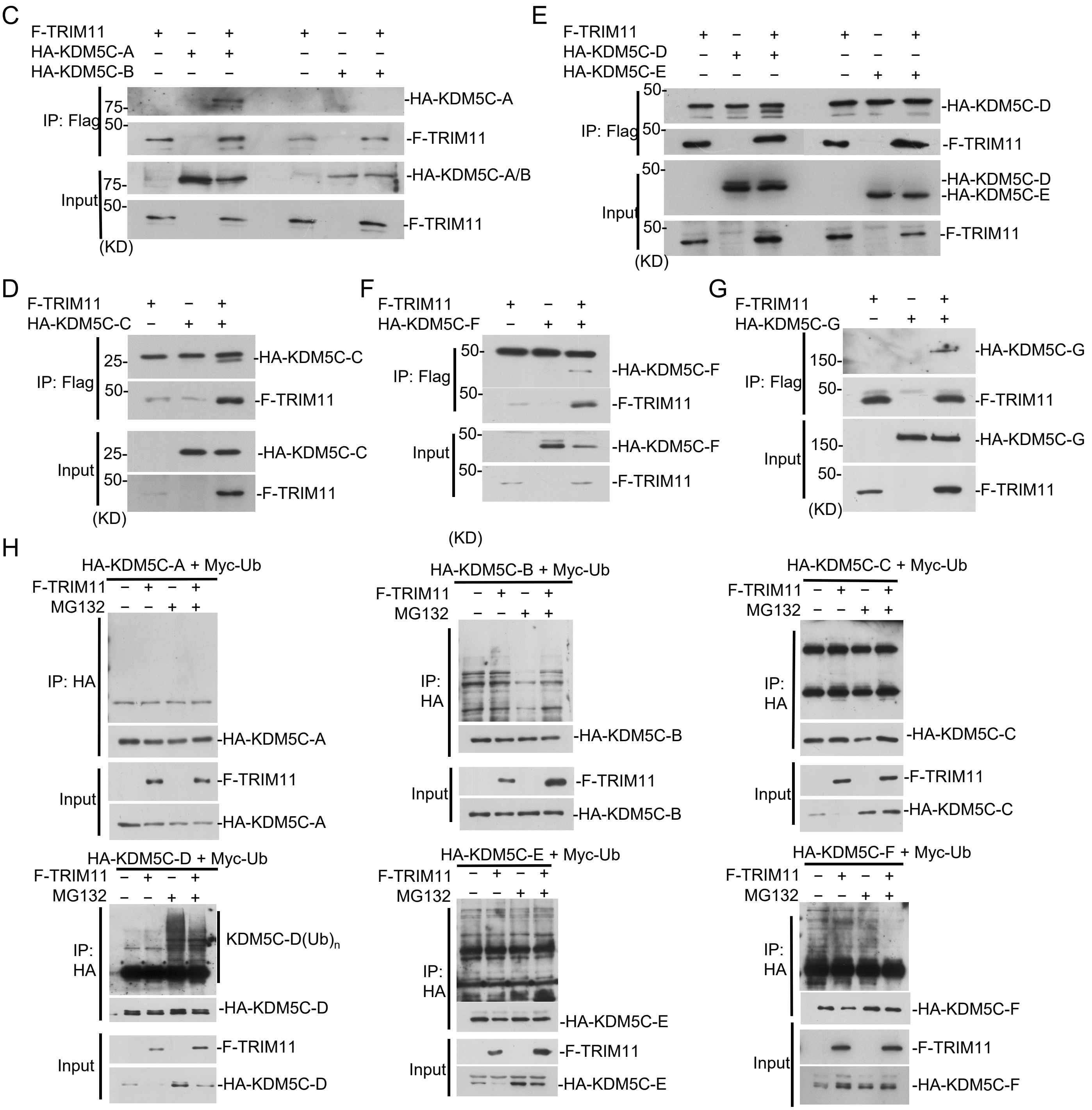

Supplemental Fig. S2 (continued)

I

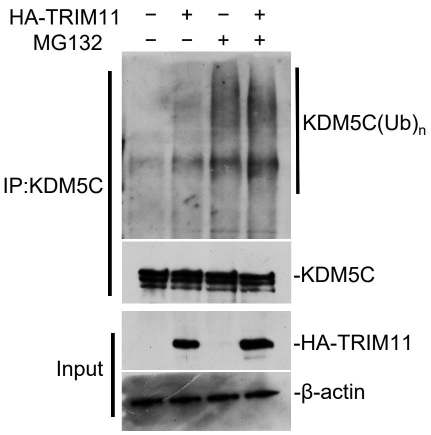

J

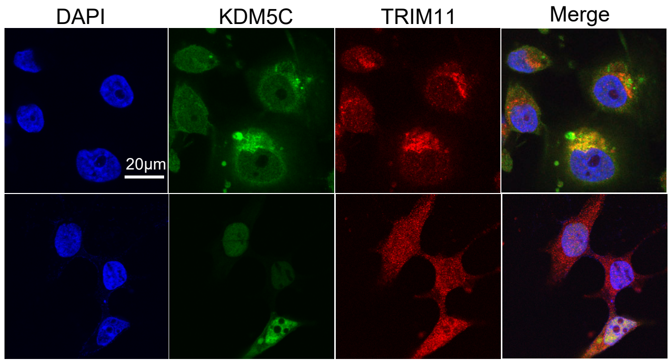

Sup. Figure S2 Poly-ubiquitination of KDM5C catalyzed by TRIM11. (A) Schematic maps of domains and truncations for KDM5C. **(B)** A summary table of the interaction and ubiquitination between TRIM11 and KDM5C truncations. **(C-G)** 293T cells were transfected with indicated plasmids for 48h and co-IP was performed with anti-Flag. **(H)** 293T cells were transfected with indicated plasmids for 36 h followed by MG132 (10 μM) treatment for 12 h. Cell lysates were subjected to IP with anti-HA followed by western blot analysis with anti-Myc. **(I)** TRIM11-overexpressed MDA-MB-231 cells were treated by MG132 (10 μM) for 12 h. Cell lysates were subjected to immunoprecipitation with anti-KDM5C antibody followed by western blot analysis with anti-Ub antibody. **(J)** Endogenous KDM5C (green) and TRIM11 (red) in MDA-MB-231 cells were assayed by immunofluorescent staining.

Supplemental Fig. S3

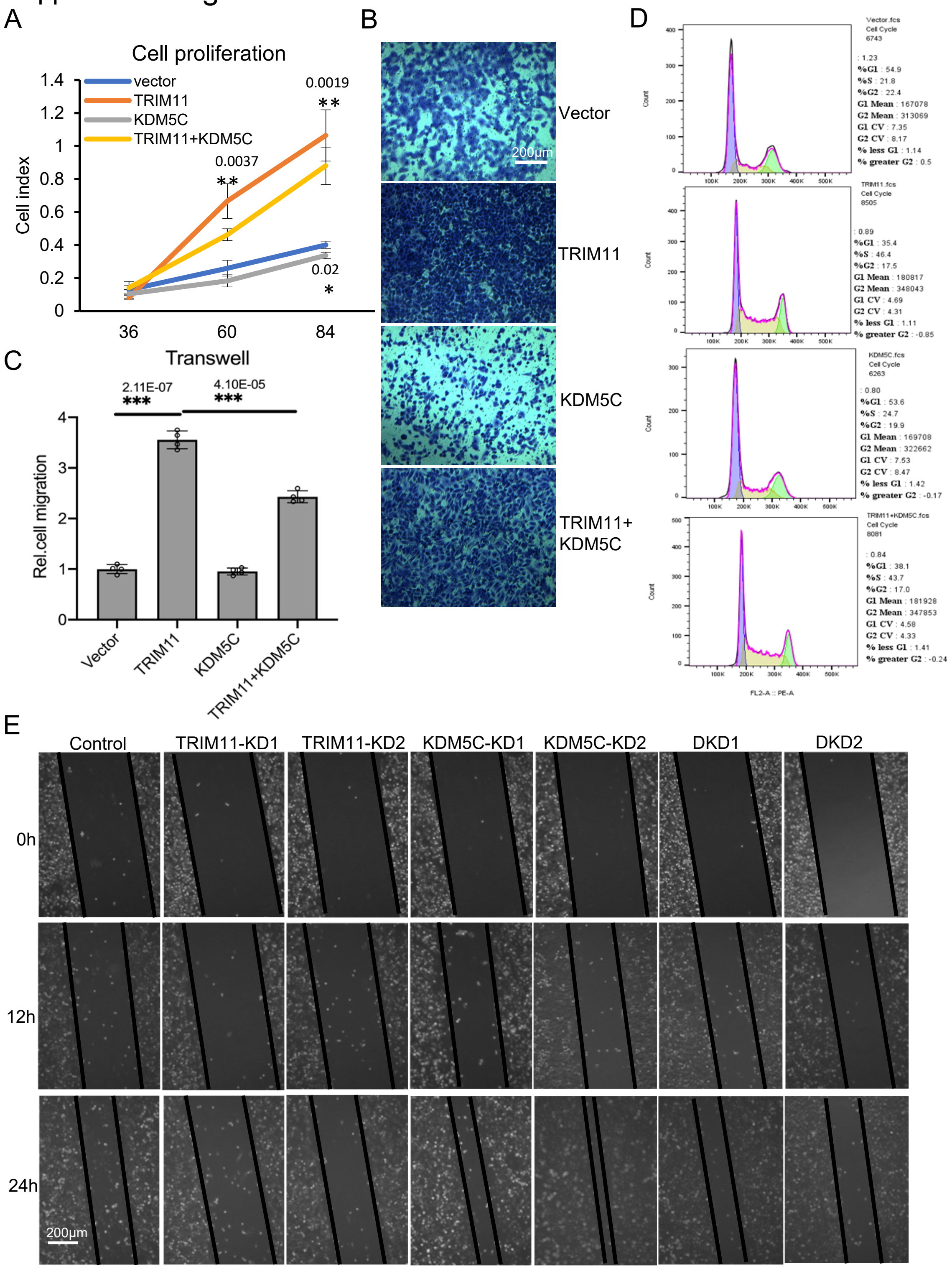

Sup. Figure S3 TRIM11 promotes cell proliferation and migration via degrading KDM5C. (A) MTT assay to show cell proliferation of the cell lines in Figure 1A. **(B&C)** Cell migration of the cell lines in Figure 1A were tested by transwell assay. Four views of the cell migration images were taken for each cell line. The number of moving cells was counted by image J. Relative cell migration was shown in (C). **(D)** The cell lines in Figure 1A were stained with propidium iodide, and then assayed by flow cytometry. Cell cycle analysis was performed on an Epics XL-MCL flow cytometer (Beck- man Coulter) with System II (version 3.0) software (Beckman Coulter). **(E)** The cell lines in Figure 3A were examined with wound healing assay. Statistics were presented as mean ± SD. * p-value < =0.05, ** p-value < =0.01, *** p-value < =0.001.

Supplemental Fig. S4

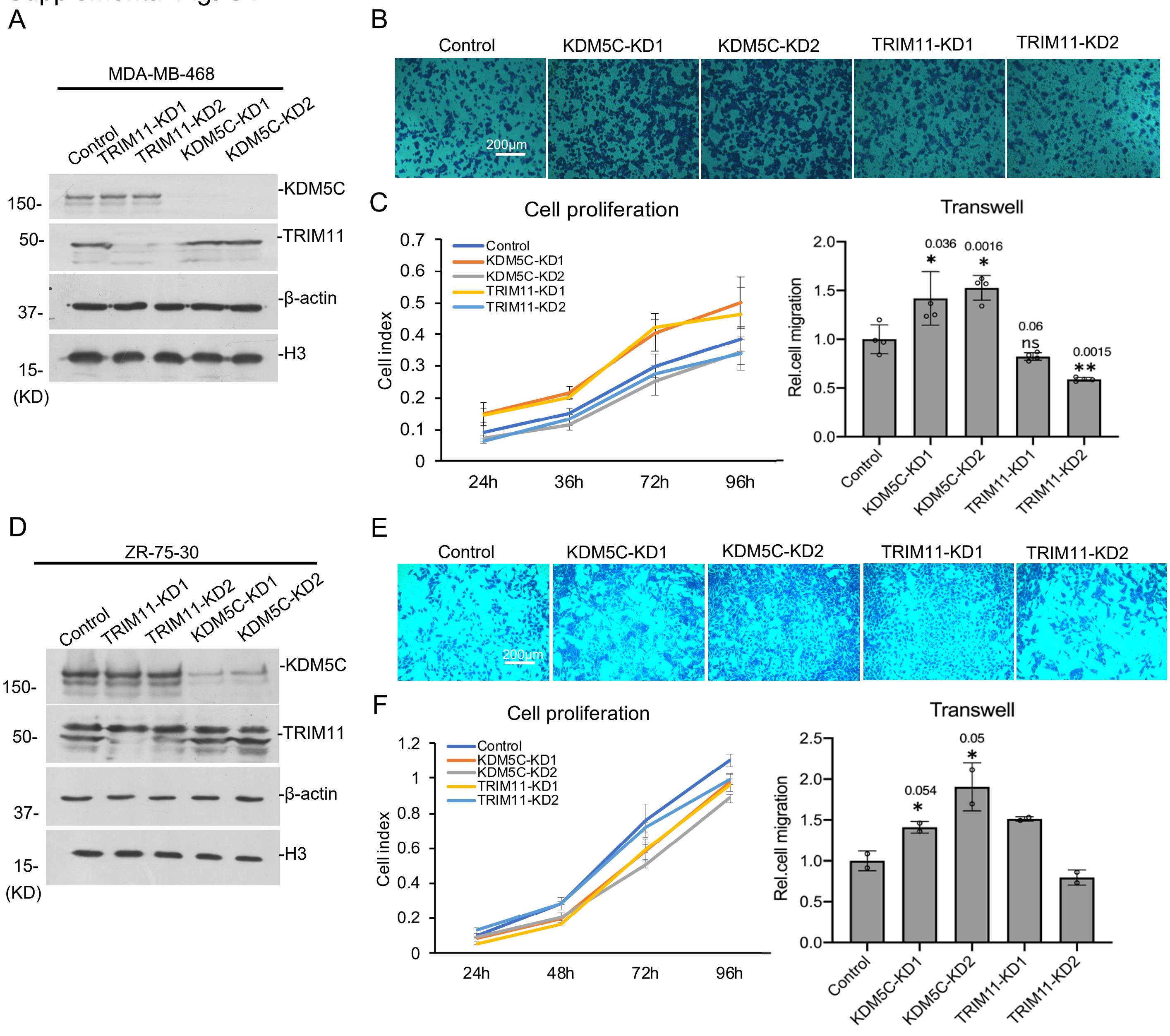

Sup. Figure S4 The functions of TRIM11 and KDM5C on cell proliferation and migration in MDA-MB-468 and ZR-75-30 breast cancer cells. (A) TRIM11-KD and KDM5C-KD MDA-MB-468 cell lines were constructed using the CRISPR/Cas9 system. **(B&C)** The cell lines in (A) were tested by transwell assay (B) and MTT assay (C). **(D)** TRIM11-KD and KDM5C-KD ZR-75-30 cell lines were constructed using the CRISPR/Cas9 system. **(E&F)** The cell lines in (D) were tested by transwell assay (E) and MTT assay (F). Statistics were presented as mean ± SD. * p-value < =0.05, ** p-value < =0.01, *** p-value < =0.001.

Supplemental Fig. S5

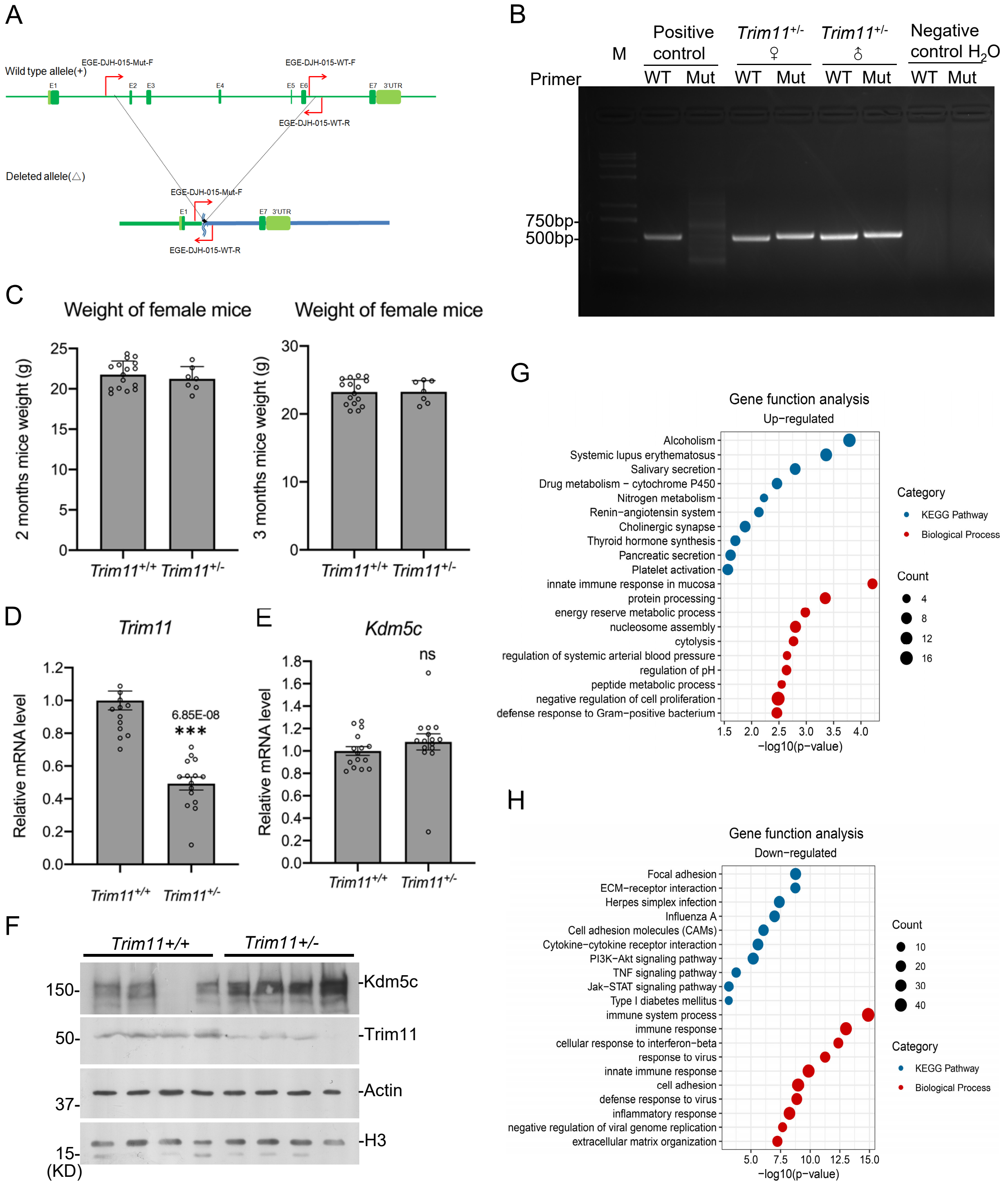

Sup. Figure S5 The function of TRIM11 in MMTV-PyVT mice. (A) Schematic diagram of *Trim11* systemic knockout mice. The gene structure of Trim11 was shown, and the exon 2-6 region was deleted based on CRISPR/Cas9 system. Two sgRNAs are designed on both sides of the target sequence, resulting in approximately ~8.5 kb of genome deletion and insertion, and deletion of exon 2-6 regions. **(B)** PCR analysis of genomic DNA of *Trim11* knockout mice. **(C)** Body weight of 2-month-old and 3-month-old female mice with or lacking *Trim11*(*Trim11*$^{+/+}$, n = 16; *Trim11*$^{+/-}$, n = 7). Statistics were presented as mean ± SD. **(D&E)** Quantitative RT-PCR analysis of *Trim11* and *Kdm5c* genes in mammary tumors of Trim11+/+ and Trim11+/- MMTV-PyVT mice (n=5, per group, 3 replicates). Results were presented as mean ± SEM**. (F)** Immunoblot analysis of Kdm5c in breast cancer tissues from Trim11+/+ and Trim11+/- MMTV-PyVT mice (n=4, per group). **(G&H)** Biological process and KEGG pathway enrichment analyses of up-regulated DEGs (C) or down-regulated DEGs (D) in Trim11+/- MMTV-PyVT mice were performed with DAVID, and items were ordered by p-value. *** p-value ≤ 0.001.

Supplemental Fig. S6

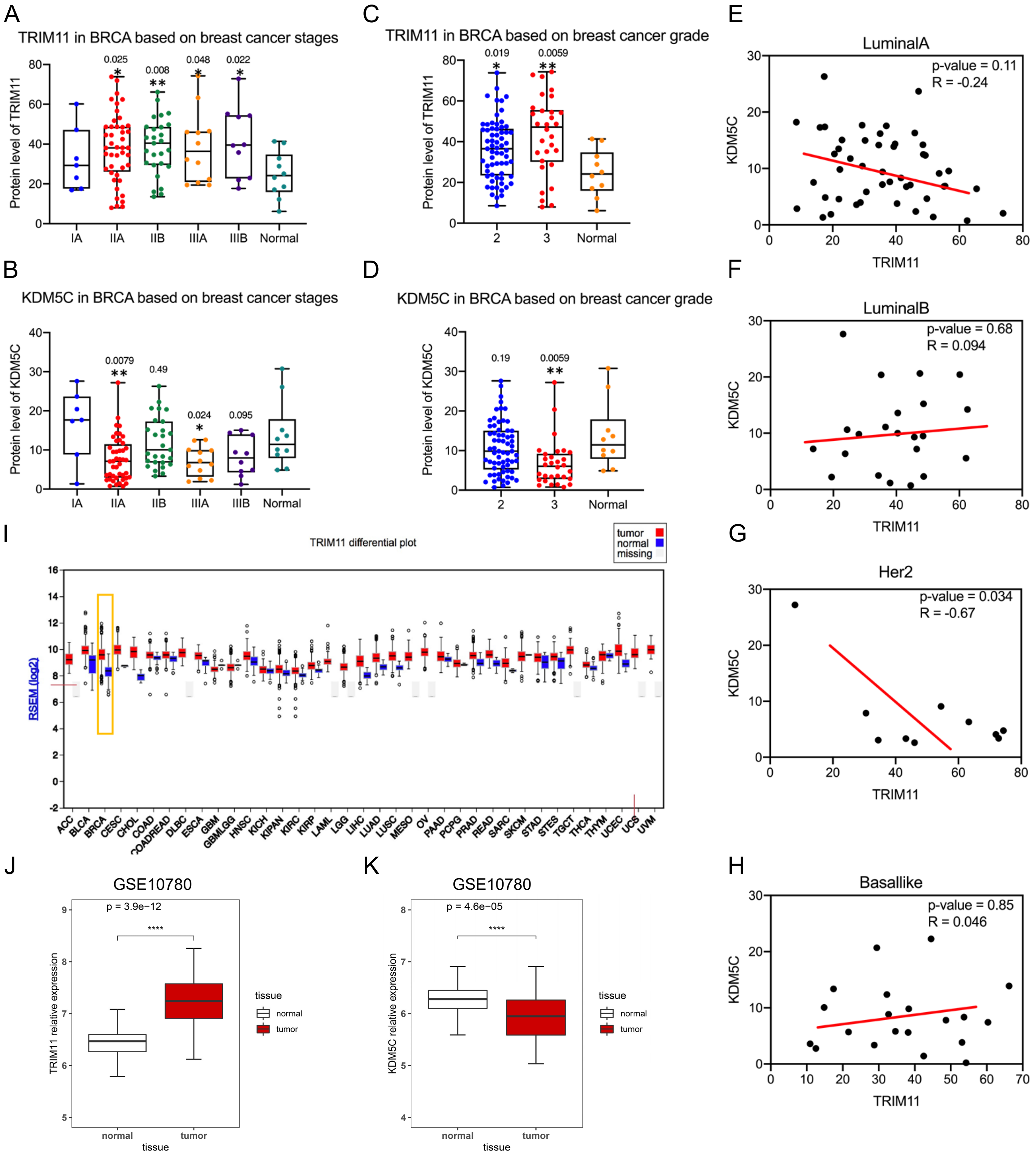

Sup. Figure S6 Correlation of TRIM11 and KDM5C in breast cancer patients. (A-D) Expression of TRIM11 and KDM5C in human breast cancer TMA was shown as mean, min to max and all points according to stages (A&B) and grade(C&D). **(E-H)** Correlation of TRIM11 and KDM5C expression level in molecular subtypes of TMA. **(I)** Boxplots showing the expression of TRIM11 in multiple tumors and normal tissues in TCGA database. **(J&K)** Expression of TRIM11 and KDM5C in human breast cancer patients was analyzed using GSE10780 data sets. * **p-value＜=0.05, ** p-value＜=0.01, *** p-value＜=0.001.**

Supplemental Fig. S7

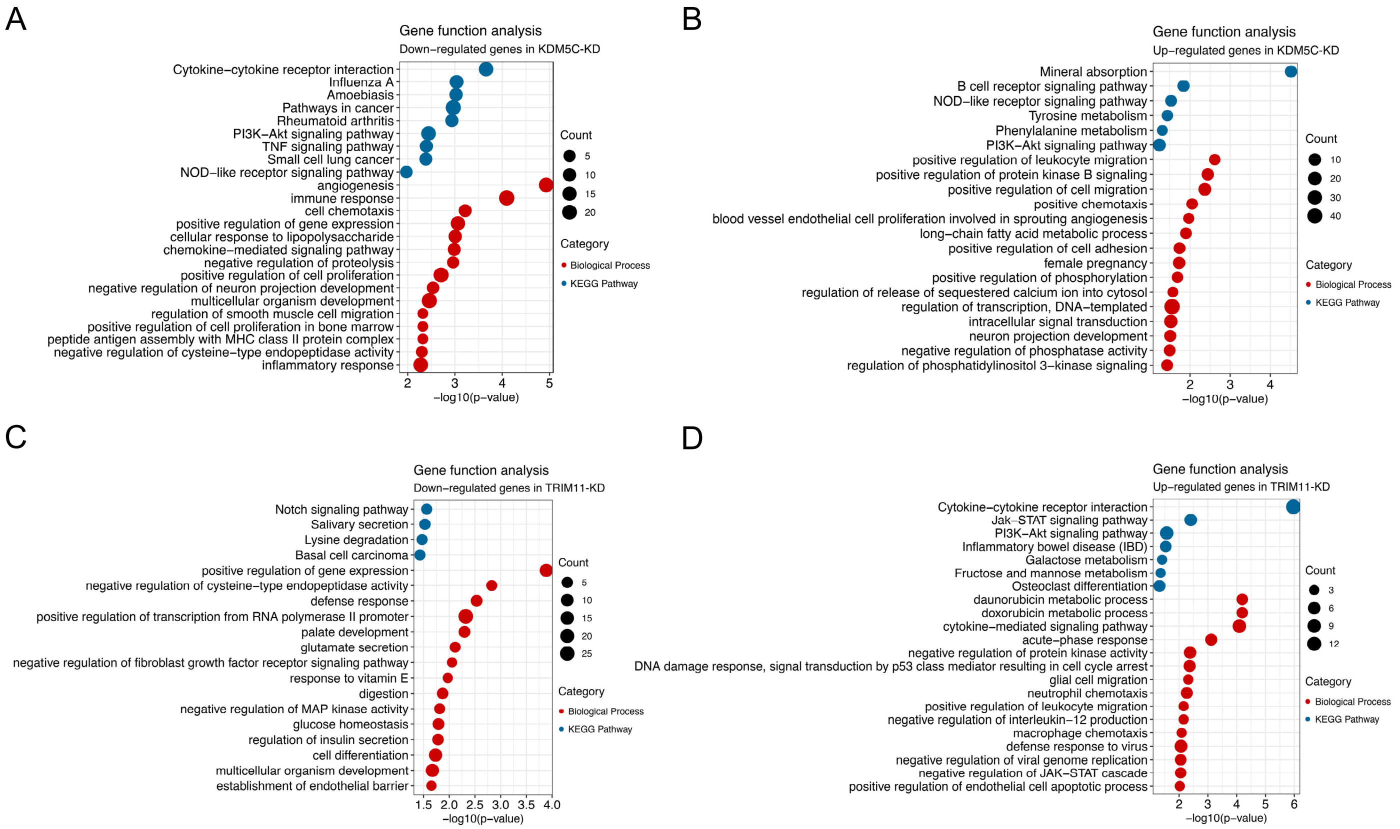

Sup. Figure S7 TRIM11 regulates H3K4me3 on enhancers through KDM5C. (A-D) Biological process and KEGG pathway enrichment analyses of DEGs down-regulated in KDM5C KD cells (A), upregulated in KDM5C KD cells.

Supplemental Fig. S8

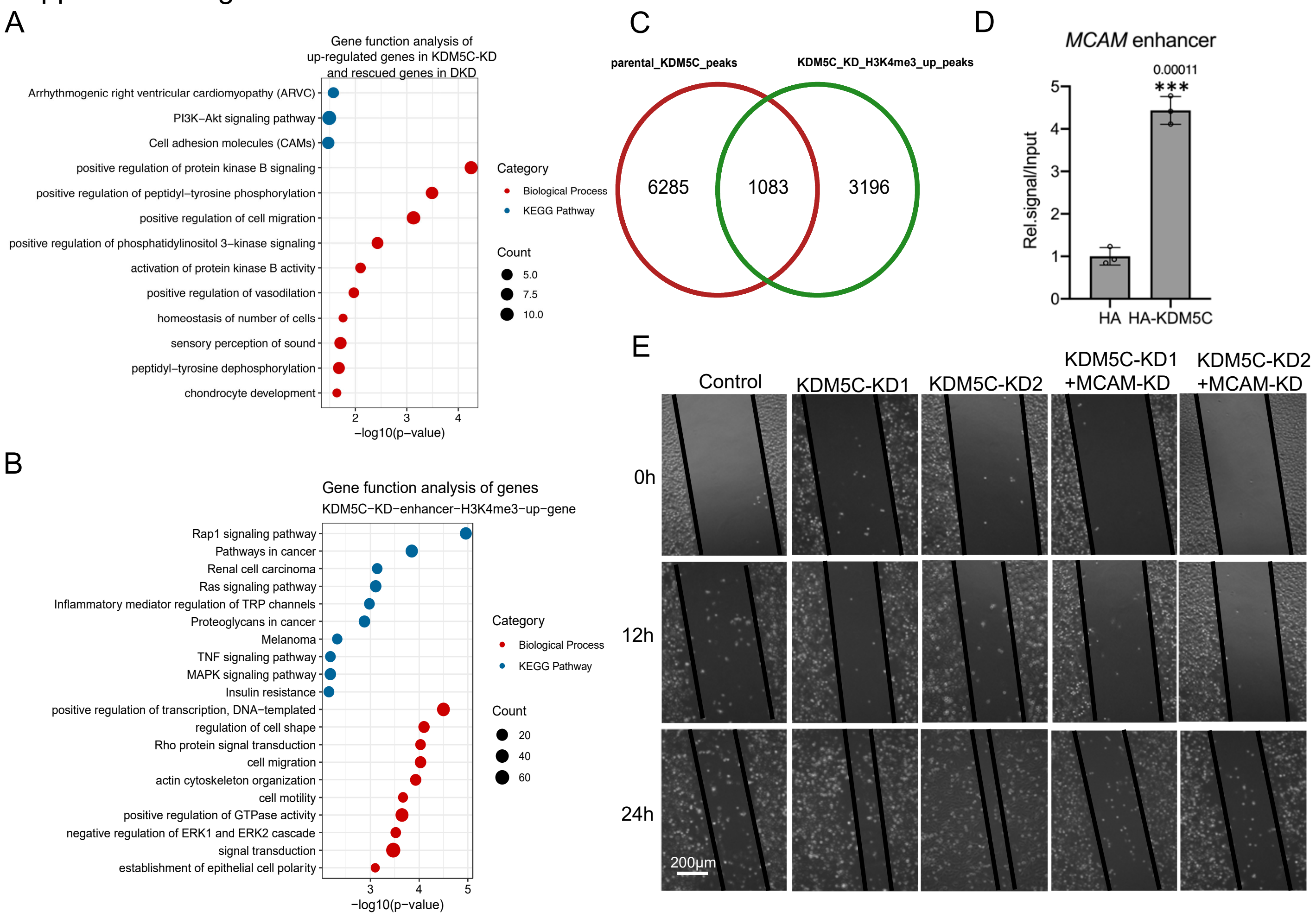

Sup. Figure S8 KDM5C targets *MCAM* enhancer and inhibits cell migration. (A) Biological process and KEGG pathway enrichment analyses of up-regulated DEGs in KDM5C-KD cell lines and rescued DEGs in DKD cell lines. Assay was performed with DAVID, and items were ordered by p-value. **(B)** Biological process and KEGG pathway enrichment analyses of adjacent genes of enhancers with up-regulated H3K4me3 in KDM5C-KD cells. **(C)** Venn Diagrams show the overlapped peaks of KDM5C occupancy (GSE71327) and up-regulated H3K4me3 on enhancers in KDM5C-KD cells (either in KDM5C-KD1 or KDM5C-KD2). **(D)** ChIP-qPCR analysis of HA-KDM5C occupancy on MCAM enhancer. Phage-HA MDA-MB-231 cell line was used as control (n = 3). **(E)** Cell migration of the cell lines in Figure 5F were tested by wound healing assay. * p-value < =0.05, ** p-value < =0.01, *** p-value < =0.001.

Supplemental Fig. S9

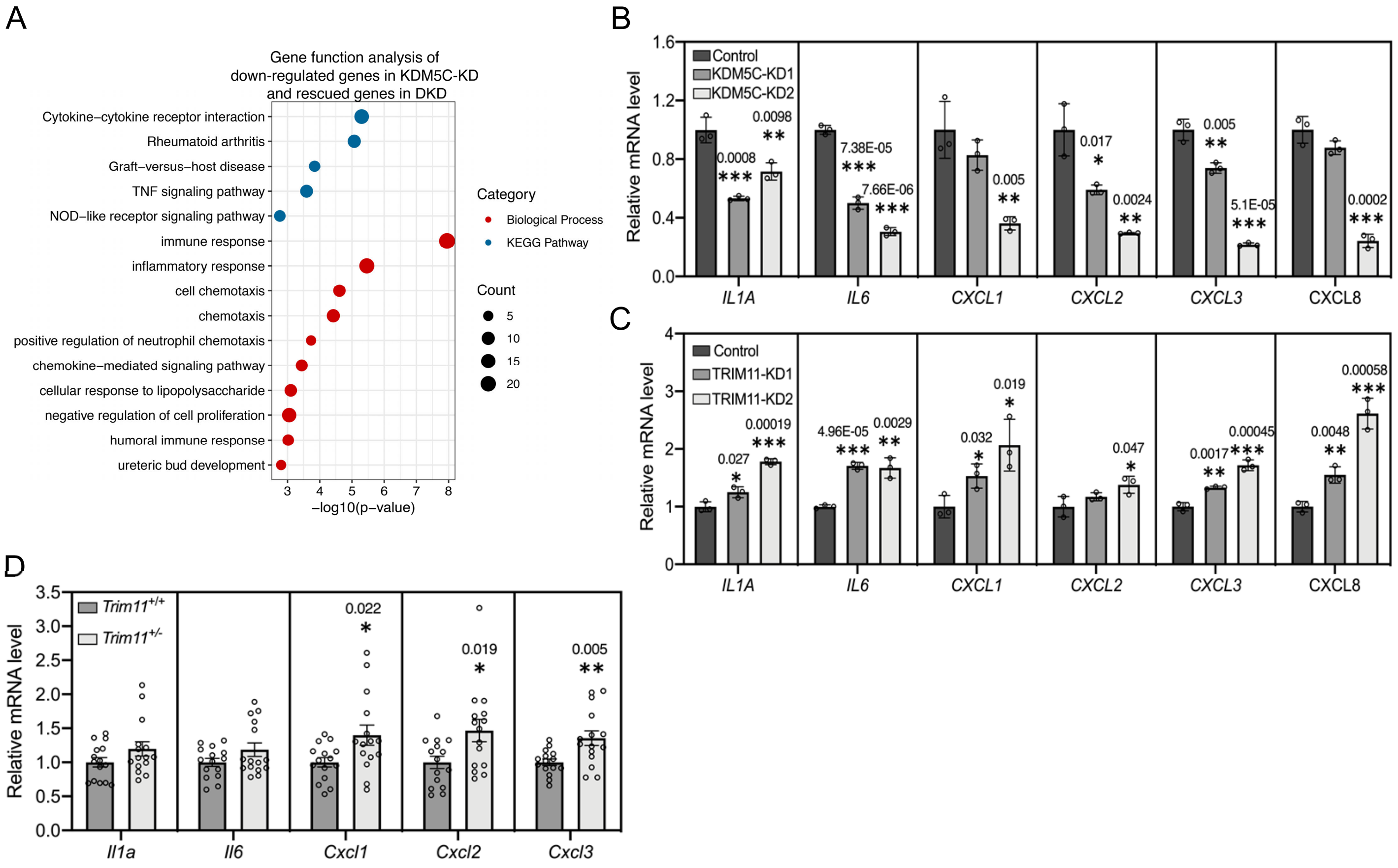

Sup. Figure S9 TRIM11 and KDM5C regulate the expression of inflammatory genes. (A) Biological process and KEGG pathway enrichment analyses of down-regulated DEGs in KDM5C-KD cell lines and rescued DEGs in DKD cell lines were performed with DAVID, and items were ordered by p-value. **(B&C)** Quantitative RT-PCR analysis of genes related to immune response in KDM5C-KD (B) and TRIM11-KD (C) MDA-MB-231 cell lines. **(D)** Quantitative RT-PCR analysis of genes related to immune response in mammary tumors of Trim11+/+ and Trim11+/- MMTV-PyVT mice (n=5, per group, 3 replicates). Statistics were presented as mean ± SD (B&C) or SEM (D). * p-value ≤ 0.05, ** p-value ≤ 0.01, *** p-value ≤ 0.001.
