# Supplementary figures and images for: Regulation of KDM5C stability and enhancer reprogramming in breast cancer
Source: Cell Death Dis. 2022 Oct 3;13(10):843. doi: 10.1038/s41419-022-05296-5 (PMC9530161; doi:10.1038/s41419-022-05296-5)

Fig1

A

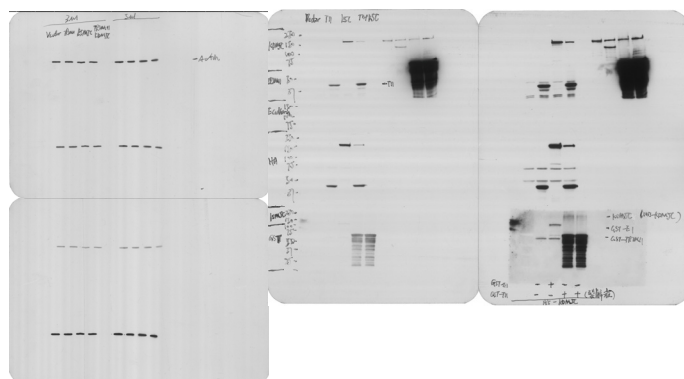

B

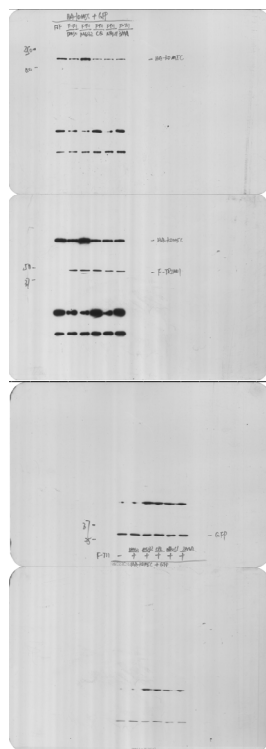

C

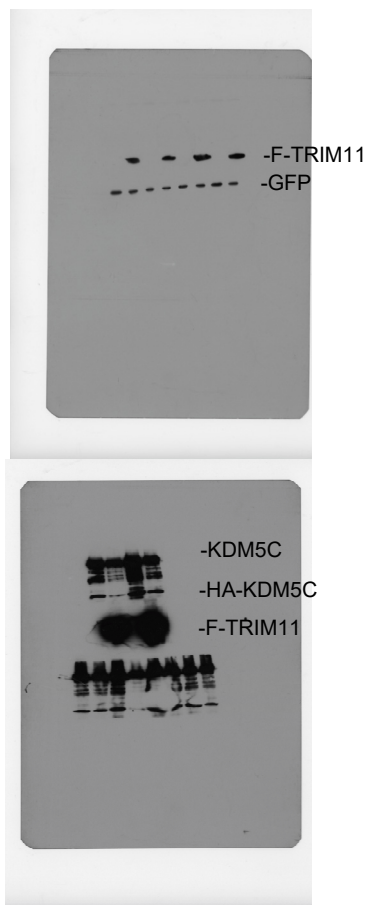

D

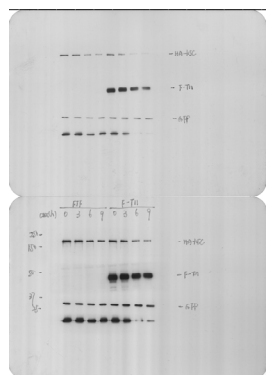

G

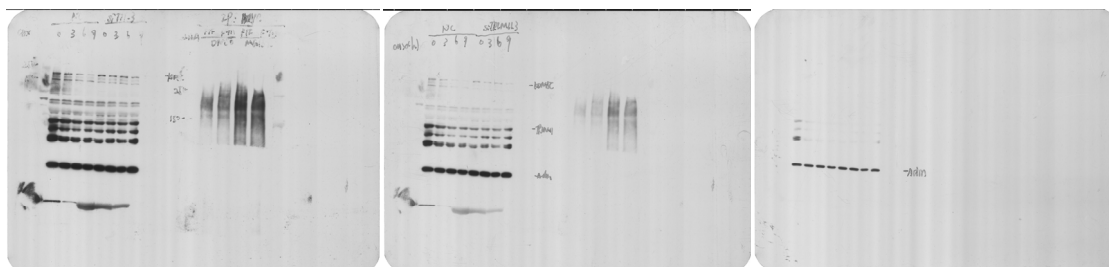

E

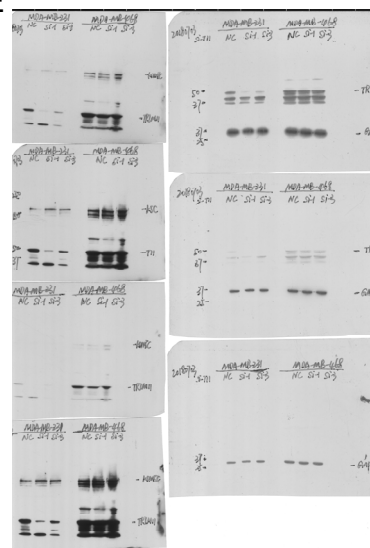

F

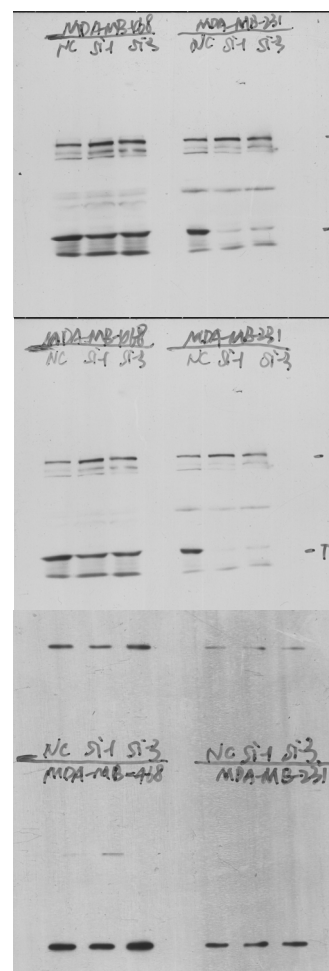

Fig2

A

B

C&D

E

F

G

H

1

J

Fig3A

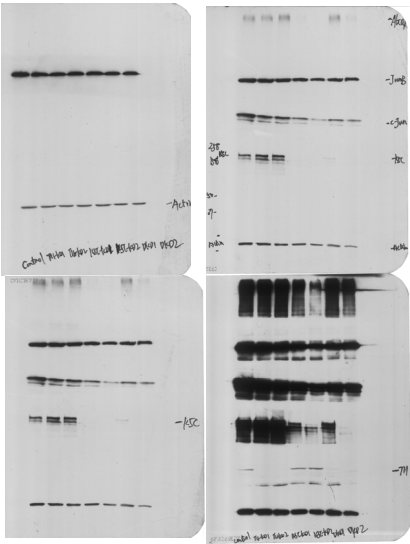

Fig3G

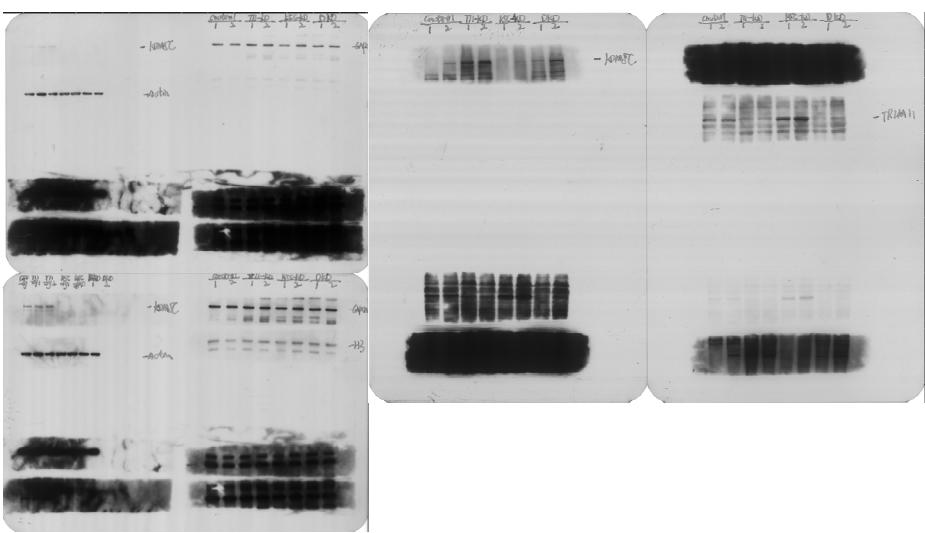

Fig.6F

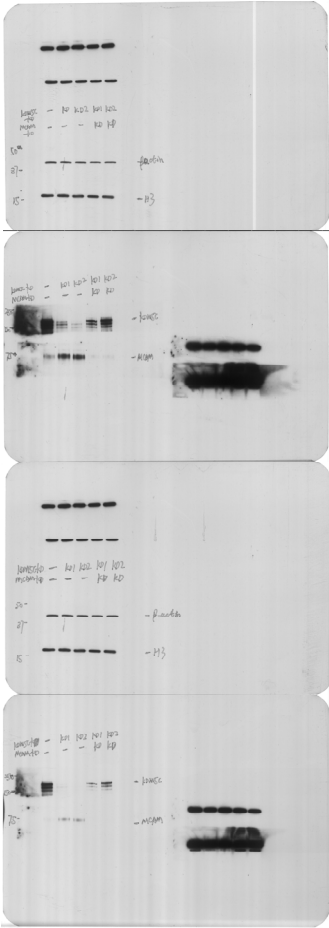

# Supplemental Fig. S1

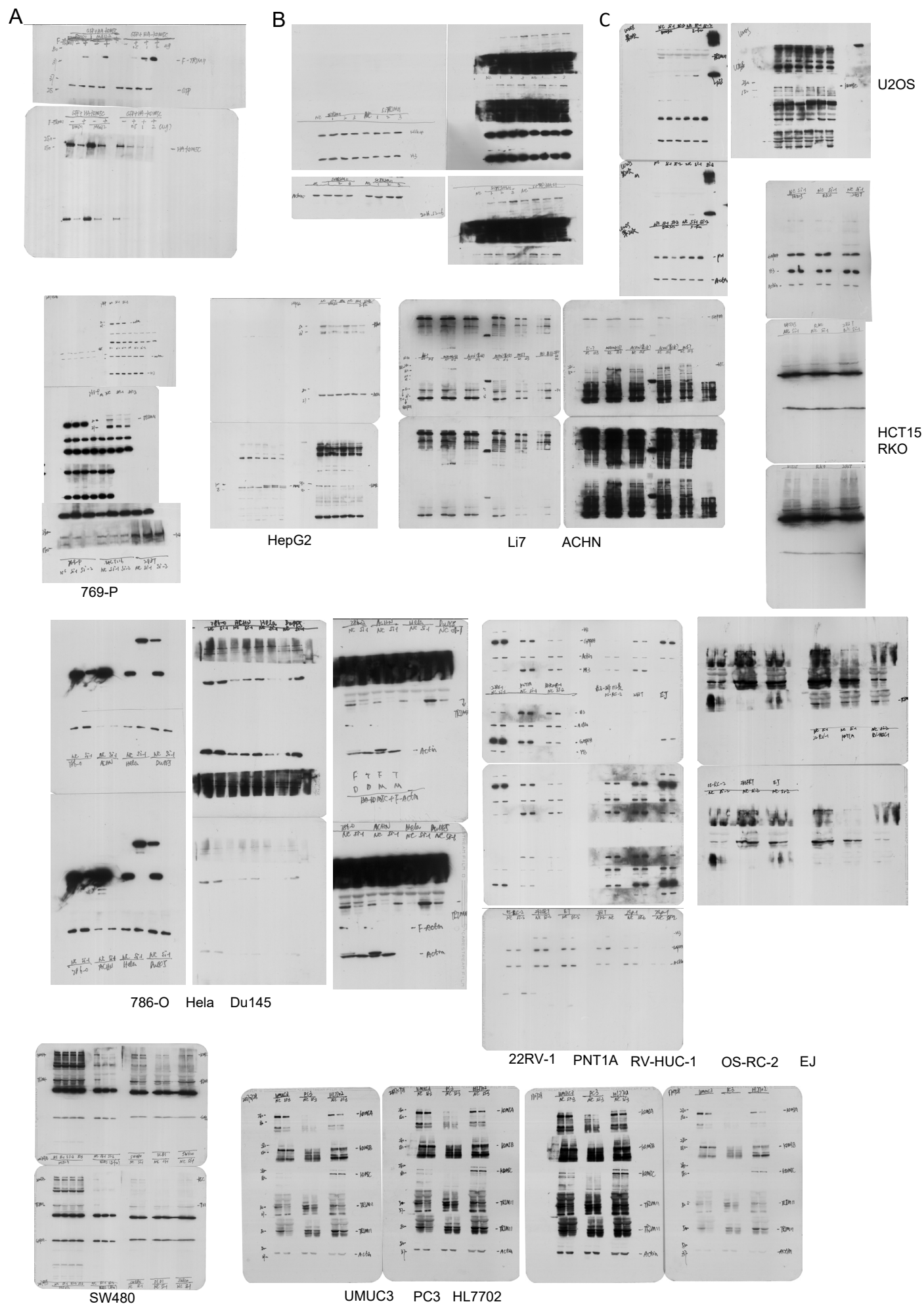

## C

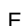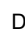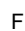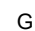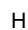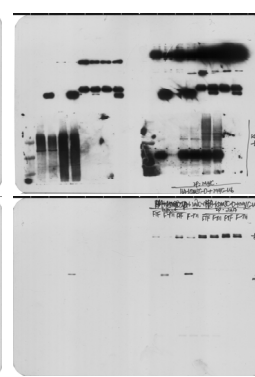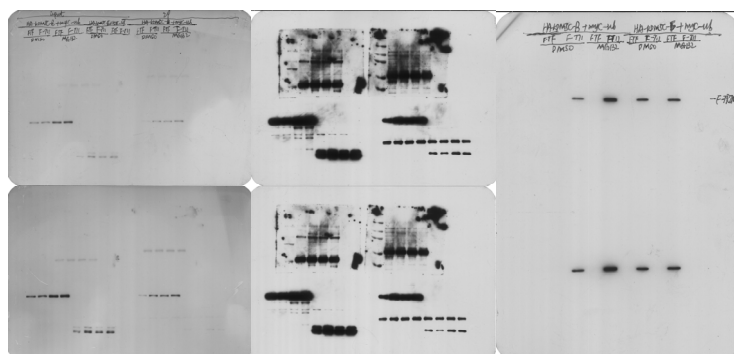

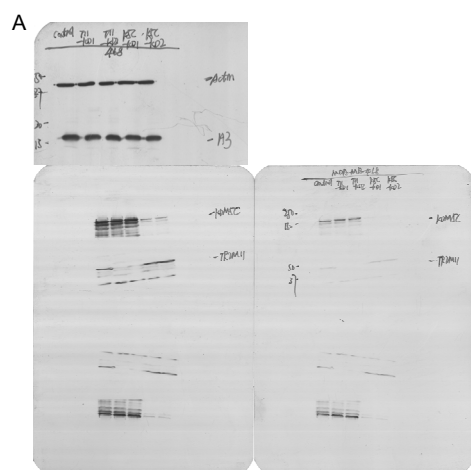

MDA-MB-468

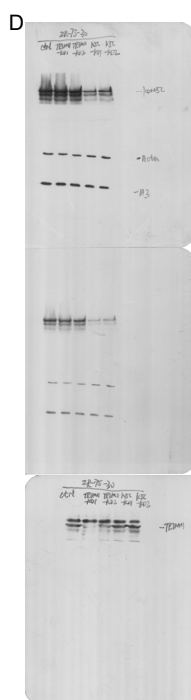

ZR-75-30

Supplement: Supplementary file 3 — Original Data File [file 41419_2022_5296_MOESM3_ESM.pdf]
